# The structure of the cereal leaf beetle (*Oulema melanopus*) microbiome depends on the insect’s developmental stage, host plant, and origin

**DOI:** 10.1038/s41598-021-99411-9

**Published:** 2021-10-14

**Authors:** Beata Wielkopolan, Krzysztof Krawczyk, Alicja Szabelska-Beręsewicz, Aleksandra Obrępalska-Stęplowska

**Affiliations:** 1 Department of Monitoring and Signaling of Agrophages, Institute of Plant Protection–National Research Institute, 20 Węgorka St, 60-318 Poznan, Poland; 2 Department of Molecular Biology and Biotechnology, Institute of Plant Protection–National Research Institute, 20 Węgorka St, 60-318 Poznan, Poland; 3 Department of Mathematical and Statistical Methods, Poznań University of Life Sciences, 28 Wojska Polskiego St, 60-624 Poznan, Poland

**Keywords:** Entomology, Microbiome

## Abstract

Cereal leaf beetle (CLB, *Oulema melanopus*, Coleoptera, Chrysomelidae) is a serious agricultural pest that causes considerable damages to agricultural production. The aim of this study was to characterize the bacterial communities associated with larvae and imagoes of CLB collected from various cereal host species and locations. The bacterial profile was characterized by 16S rRNA gene sequencing at the V3-V4 hypervariable region. Using taxonomy-based analysis, the bacterial community of CLB containing 16 phyla, 26 classes, 49 orders, 78 families, 94 genera, and 63 species of bacteria was identified. The abundance of *Wolbachia*, *Rickettsia,* and *Lactococcus* genus was significantly higher in CLB imagoes than in larvae. Statistical analysis confirmed that the bacterial community of the larvae is more diverse in comparison to imagoes and that insects collected from spring barley and wheat are characterized by a much higher biodiversity level of bacterial genera and species than insects collected from other cereals. Obtained results indicated that the developmental stage, the host plant, and the insect’s sampling location affected the CLB’s microbiome. Additionally, the CLB core microbiome was determined. It consists of 2 genera (*Wolbachia* and *Rickettsia*) shared by at least 90% tested CLB insects, regardless of the variables analysed.

## Introduction

Insects are a major component of biodiversity in virtually all terrestrial ecosystems, making them very important for environmental impact assessment^1^. Coleoptera is the most species-rich and diversified order of insects in the world and includes over 360,000 species^2^. Although many coleopteran insects are serious agricultural pests and cause considerable damages to agricultural production, little information is available on the bacteria associated with them. So far, the microbiome has been examined in a few coleopteran species, including dung beetle (*Onthophagus taurus*, Coleoptera: Scarabaeidae)^3^, mountain pine beetle (*Dendroctonus ponderosae,* Coleoptera, Curculionidae)^4^, Asian long-horned beetle (*Anoplophora glabripennis*, Coleoptera, Cerambycidae)^5^. As yet, there is no information on the microbiome of serious cereal pest—*Oulema melanopus* (cereal leaf beetle, CLB, Coleoptera, Chrysomelidae), except for *Wolbachia* and *Rickettsia,* which presence in CLB was previously mentioned^6^. The CLB has a wide host range, including wheat, barley, oat, and rye. Beetles of CLB may also feed on corn, sorghum, sudangrass, and grass weeds (e.g., wild oats, quackgrass, timothy, annual and perennial ryegrass). The first symptoms of CLB activity are damages of plant foliage caused by their adults feeding. CLB injury is characterized by elongated, slender slits in the upper leaf surface. Both adults and larvae damage cereal plants by feeding on the leaves, but the larvae are considered to be more harmful than imagoes and are the target of insecticide control. Larvae feed all the green material down to the lower cuticle (an elongated windowpane in the leaf) (https://www.ag.ndsu.edu/publications/crops/north-dakota-small-grain-insects-cereal-leaf-beetle-oulema-melanopus-l-coleoptera-chrysomelidae). Their feeding causes considerable quantitative and qualitative losses in the crop yield.

The microbiome of many insects has a significant impact on insects' physiology and fitness^7^. Some bacterial symbionts are necessary for the growth, survival, and reproduction of their insect host through their influence on the insect’s digestive processes^8,9^. Other bacterial symbionts are not essential but can have a significant impact on many aspects of an insect’s life, such as detoxification of insecticides^10^ or plant secondary metabolites^11^, immunity, speciation, and defense against predators^12–14^, entomophages and entomopathogens^15^, tolerance to environmental stresses^16^ or adaptation to specific host plants^17^. The change in the bacterial structure can affect insect’s functions associated with development, fecundity, metabolism, immunity, and susceptibility to pathogens. Therefore, bacterial symbionts can change the pest status of insects, triggering outbreaks in some insect species, but also reducing the densities of others insects^2^.

Insects have an open circulatory system that allows hemolymph to flow throughout the body, therefore microbial communities are colonizing many insect tissues^18,19^. Insects symbiotic bacteria colonize not only the gut, but can be found in hemocoel^2^, body cavity^9^, or salivary glands, and thus can be present in insect oral secretion. Some insects have a specific symbiotic organ—bacteriome composed of bacteriocytes that harbour symbiotic bacteria^20,21^. The insect’s microbial diversity depends on a variety of conditions found within the insect body, including oxygen conditions (aerobic, anaerobic), pH^18,19,22^, or a relationship with insect^18,19^. It was indicated that the composition of microbes also depends on the insect’s developmental stage^23^, host taxonomy^24^, environment^25^, diet type^26^, or social interactions between insects^27^.

Symbionts can be transmitted vertically, horizontally, or by the combination of both ways^28^. Despite, the radical changes of the bacterial community taking place during the metamorphosis, numerous holometabolous species (CLB is one of them) vertically transmit their symbionts. Larva and adult stage can harbour the same symbionts but in different tissues or may differ in bacterial composition due to the metamorphosis or differences in habitat or diet. Likely, the microbes can be acquired by insects from the environment due to their ubiquitous occurrence in soil and plants^2^. Insects can acquire symbionts from plants during feeding^29^. Bacteria acquired via the diet or soil can be also involved in many life processes of insects^30,31^.

Insects cause damage directly by wounding plants by feeding, and indirectly by transmitting plant pathogens that can develop disease symptoms^32^. Nowadays, to control insects pests, several approaches can be used: chemical insecticides, the sterile insect technique (SIT), or biological pest control, using predators and parasitoids^33^. Some crop pests, including CLB, have evolved detoxification mechanisms against the insecticides used^10,34,35^. Insects exhibit complex symbiotic interactions with microbes, which can provide resources for developing species-specific pest management tactics^14,36,37^, therefore insect-associated bacteria, especially symbionts, are gaining more and more attention as a promising tool in insect pest management^33^.

The objective of this research was to (i) characterize the CLB bacterial community, (ii) determine whether plant host (here cereals), locations from which insects were collected and insect’s developmental stage affect the CLB microbiome composition and diversity, and (iii) determine the CLB core microbiome, defined as the most common and shared microbes for the majority of the analysed CLB insects, regardless of the variables tested, that may be involved in life processes vital for the CLB.

## Material and methods

### *Oulema melanopus* sample collection

Larvae and imagoes of CLB rearing on the seven kinds of cereal crops: triticale (*Triticosecale*), winter and spring wheat (*Triticum*), winter and spring barley (*Hordeum*), oat (*Avena*), and rye (*Secale*) were collected from three geographically distinct locations (Winna Góra (52°12′17″N 17°26′48″E), Zybiszów (51°03′50″N 16°54′41″E), and Kościelna Wieś (51°47′08″N 18°00′34″E), in 2018–2019. The meteorological conditions during insect development and sampling were comparable except for rainfalls (Supplementary Table 3S). The insect material had been collected with the prior station's approval. Together three variables, including developmental stage (imago and larva), host plant (7 cereals), and location (3 locations) were analysed. Insects were placed in a sterile 1.5 ml Eppendorf tube, filled with 70% ethanol, and stored at − 20 °C until DNA isolation.

### DNA extraction from insects

The study was based on 100 samples of CLB insects, divided into 20 sample sets. Each set consisted of 4 samples containing DNA isolated from a single insect and one sample constituted the DNA pooled together from 3 insect individuals (Supplementary Table 1S). In total, 98 CLB larvae and 42 imagoes were used for the study. Prior to DNA extraction, each insect was washed in 70% ethanol and subsequently rinsed two times with sterile distilled water to remove environmental contaminants. DNA was individually extracted from the whole specimens of CLB of larvae and imagoes by using the Genomic Mini AX Bacteria kit (A&A Biotechnology) according to the manufacturer’s instructions with RNase A and Proteinase K treatment step. DNA was resuspended in 30 µl of 5 mM Tris/HCl, pH 8.5. DNA quality and concentration were assessed using a Nano-Drop ND-1000 spectrophotometer (Thermo Fisher Scientific). The DNA integrity was checked by running the 0.8% agarose gel electrophoresis. The extracted DNA was stored at − 20 °C until the sequencing of 16S rRNA. The DNA extraction process was verified against the presence of 16S rDNA by PCR using bacterial universal primers^38^ to exclude potential contaminants occurring in samples due to isolation process^39^, as well as commonly reported^40^ in NGS projects contamination with *Cutibacterium acnes* using the nested-PCR technique^41^. No bacteria other than *Wolbachia* and *Cutibacterium* were identified in all analysed samples.

### Bacterial sequencing

The identification of CLB-associated bacteria was done by 16S rRNA gene sequencing at the V3-V4 hypervariable region by using Next Generation Sequencing (NGS). Sample quality control was done with Qubit dsDNA BR or HS (ThermoFisher). Separate amplicon libraries were prepared for each of the 100 samples by using Quick-16S NGS Library Prep Kit (Zymo Research) according to the manufacturer’s protocol. The sequencing was done with 20 ng of DNA per sample (MiSeq Illumina) with read length 2 × 250 bp, output 25 K clusters per sample. Amplicon libraries targeting the V3–V4 hypervariable regions of the 16S rRNA gene were generated (CeGat GmbH, Germany). The positive and negative controls of the sequencing process were also done. After library preparation, both controls were checked and there were not any abnormalities: negative control was negative (no measurable DNA) and positive control was positive (expected DNA amount and expected fragment length).

### Bioinformatic processing

The reads were filtered for containing unknown nucleotides (Ns) and low-quality bases before trimming and merging using the R package DADA2^42^. Sequences that mapped to chloroplast or mitochondrial DNA were excluded. The resulting fasta files were used for taxonomic classification. Assigning the taxonomic labels to DNA short reads was done by examining the k-mers within a read and querying a database with those k-mers, using the Kraken2 algorithm implemented in OmicsBox software (v. 1.4.12). The k-mers within the tested reads were mapped to the Kraken’s genomic library to the lowest common ancestor (LCA) in the taxonomic tree of all genomes that contain that k-mer. Finally, the set of LCA taxa that correspond to the k-mers in a read were analyzed to create an operational taxonomic unit (OTU) (http://manual.omicsbox.biobam.com/user-manual/module-metagenomics/taxonomic-classification/). In our study, Kraken2 results were filtered using a confidence threshold of 0.05 (http://www.biobam.com/omicsbox). For statistical analyses the singletons, defined as the taxa observed in less than 2 samples, were excluded.

### Microbial composition analyses

In order to verify which factors influence the microbial composition on each taxonomic level, we first modeled the abundance of bacterial community measured by a normalized number of reads, with respect to three following variables: the insect’s developmental stage, cereal plant host, and location from which insects were collected. Above mentioned variables were included as fixed effects. In addition, the taxonomical rank was included as the random intercept. To assess the appropriateness and fit of the model, several procedures were performed. Hurdle negative binomial generalized linear mixed model (hNB GLMM) was compared with the negative binomial generalized linear mixed model (NB GLMM) as well with hurdle negative binomial model where rank taxa were not included (hNB GLM). The comparison with the hurdle model with rank taxa included as the fixed effect was also considered. However, due to too many levels for rank taxa variable this model could not be assessed. To verify the appropriateness of the choice of hurdle model and evaluation of estimation of parameters simulation-based tests were performed for over/underdispersion and zero-inflation with the usage of R package DHARMa^43^. The models could be fitted with the usage of functions implemented in the R package glmmTMB^44^. The fit of the hNB GLMM model was checked with the usage of the conditional r-squared value calculated based on the methodology described by Nakagawa and implemented in R package performance^45,46^. The importance of variables was calculated based on the methodology described by Biecek and Burzykowski^47^. The permutational procedure that measures penalty if the effect of a selected variable is removed was applied. Based on the suggestion in Biecek and Burzykowski (chapter 15.3.4) as a goodness of fit measure for the calculations, Pearson’s statistic was chosen. In addition, the influence of the analysed variables was determined with the Wald type II test^48^. Next for the fixed effects, we performed multiple comparisons procedure to obtain the information on which pairs of levels of each variable: the insect’s developmental stage, location, and plant host resulted in significant differences between mean values of bacterial abundance.

We followed the methodology described by the authors of the R package animalcules^49^, which performs analysis based on summed up OTUs within the taxonomic level. Next, to verify the influence of the random effect, we calculated the 99.9% confidence intervals for intercept for each analysed taxa. By doing this, we obtained the information for which taxa the abundance is significantly different from the overall mean abundance for all taxa. This set of taxa was denoted as Top Biome.

For the bacterial taxa listed as the Top Biome, the abundance values for the insect’s developmental stage and host plant at phylum, class, order, family, and genus level were calculated. The average abundance of the considered taxa based on the Top Biome for the above-mentioned taxonomic levels was presented using the Sankey diagram available in R package ggalluvial^49^. The Sankey diagram was additionally used to present the relation of the abundances between developmental stages and host plants for the most abundant bacterial genera and species from Top Biome. In the case of the Sankey diagram for bacterial genera and species, the abundances for each group were calculated as an average based only on samples with non-zero relative abundance. The abundance was also presented in the barcharts.

The heatmap based on hierarchical clustering with Ward method^50^ for the relative abundance of the 10 species and 10 the most abundant genera from Top Biome in relation to the CLB developmental stage, host plant and location from which the insects were collected was also prepared using R package pheatmap^51^.

### Differential abundance analysis

Differential abundance analysis with the usage of DESeq^52^ method implemented in R package animalcules^49^ was performed to discriminate bacteria at different taxonomic levels between insect samples depending on the insect’s developmental stage and cereal plant host to assess which taxa were responsible for the observed differences.

### Biodiversity analysis

Bacterial biodiversity was assessed with several identifiers. The first method concerns the alpha biodiversity, which describes the richness and equality of the microbial community in the sample. For this analysis, the Shannon index of dissimilarity^53^ was used. Based on this measure a parametric Welch two-sample T-test^54^ for variables with two levels (the insect’s developmental stage) was performed, whereas Kruskal Wallis test^55^ was used for variables with more than two levels (cereal plant host and locations).

Permutational Multivariate Analysis of Variance (PERANOVA)^56^ available in vegan package^57^ was performed for beta diversity, which allows for the determination of significant differences in the level of biodiversity measured by the beta coefficient. The distance matrices needed for the procedure were calculated using the Jaccard’s index^58^, Bray–Curtis index^59^, and Chao similarity index^60^. The obtained p-values were calculated in permutational procedure with 1000 permutations.

The richness estimators for the insect’s developmental stage, cereal plant host, and location were separately calculated using First-order jacknife Estimator^61^ that calculates genera and species diversity present in a dataset and is available in fossil package^62^.

Furthermore, the relations between levels of bacterial diversity for the insect’s developmental stage, host plant, and location were determined. The bacterial genera and species occurring in imagoes, in at least one sample, were analysed. Similarly, the set of bacterial genera and species observed in at least one sample was established for larvae. Next, the logical relations between sets of bacterial genera and species for larvae and imagoes were designated and presented in a form of Venn diagrams available in R package venn^63^. Analogously, the Venn diagram for the cereal hosts and locations was prepared. For the exploration of sample similarities and variance, Principal Coordinate Analysis available in R package ape^64^ with Jaccard’s index^58^ was also used.

### Core microbiome determination

For each level of each variable, the percentages of samples with positive relative abundance were calculated for each bacterial genera and species. To determine the core microbiome only bacteria available in at least 90% of CLB individuals tested in each group were taken into consideration. The shared bacteria regardless of the tested variable were defined as the core microbiome and presented using the Venn diagram.

All statistical analyses were carried out using R software 3.6.2^65^. All visualizations were prepared with ggplot2 and RColorBrewer packages^66,67^.

## Results

### The structure of the CLB microbiome. Relative abundance of bacteria at different taxonomic levels

A total of 11,803,636 raw reads were generated from all insect samples. After quality trimming, filtering, and downstream analyses, 3,953,747 high-quality sequences were obtained. An average of 39,537 high-quality reads per insect sample was generated in the variable regions of the 16S rRNA gene. The number of reads varied between particular insect samples (max. 93.909, min. 9.220) (Supplementary Table 2S). The sequence data were submitted to the SRA database (https://submit.ncbi.nlm.nih.gov/about/sra/) under the BioProject ID: PRJNA753682. Bacterial taxonomy was determined using Kraken2 database (OmicsBox, v. 1.4.12). Obtained OTUs were summed within taxonomic rank, and assigned to 16 bacterial phyla, among them 26 classes, 49 orders, 78 families, and 94 genera of bacteria were identified (Table 1 and Supplementary Table 4S). *Cutibacterium acnes* presence was found at low levels in the majority of tested samples but excluded from further analyses because it was identified also in the negative control of the extraction process.

**Table 1:** Lists of bacterial taxa classified at phylum, order, and genus level.

Phylum	Order	Genus
Acidobacteria	Bryobacterales	*Candidatus Solibacter*
Actinobacteria	Acidimicrobiales	*Ilumatobacter*
	Actinomycetales	*Actinomyces*
	Corynebacteriales	*Corynebacterium*
		*Lawsonella*
		*Rhodococcus*
	Micrococcales	*Brevibacterium*
		*Frondihabitans*
		*Microbacterium*
		*Arthrobacter*
		*Kocuria*
		*Sanguibacter*
	Propionibacteriales	*Aeromicrobium*
		*Friedmanniella*
		*Nocardioides*
		*Microlunatus*
		*Pseudonocardia*
	Streptomycetales	*Streptomyces*
Alphaproteobacteria	Rickettsiaceae	*Rickettsia*
Bacteria	Vicinamibacteria	*Luteitalea*
Bacteroidetes	Bacteroidales	*Paludibacter*
		*Porphyromonas*
	Cytophagales	*Spirosoma*
		*Hymenobacter*
		*Pontibacter*
	Flavobacteriales	*Flavobacterium*
		*Chryseobacterium*
		*Cloacibacterium*
	Sphingobacteriales	*Mucilaginibacter*
		*Pedobacter*
		*Sphingobacterium*
Cyanobacteria	Nostocales	*Anabaena*
Firmicutes	Bacillales	*Bacillus*
		*Geobacillus*
		*Terribacillus*
		*Gemella*
		*Listeria*
		*Paenibacillus*
		*Staphylococcus*
	Lactobacillales	*Aerococcus*
		*Carnobacterium*
		*Dolosigranulum*
		*Enterococcus*
		*Vagococcus*
		*Lactobacillus*
		*Leuconostoc*
		*Limosilactobacillus*
		*Weissella*
		*Lactococcus*
		*Streptococcus*
	Eubacteriales	*Lachnoclostridium*
		*Peptostreptococcaceae*
	Selenomonadales	*Selenomonas*
	Veillonellales	*Dialister*
		*Veillonella*
	Tissierellales	*Anaerococcus*
		*Finegoldia*
		*Peptoniphilus*
Fusobacteria	Fusobacteriales	*Fusobacterium*
Nitrospirae	Nitrospirales	*Nitrospira*
Proteobacteria	Caulobacterales	*Brevundimonas*
		*Caulobacter*
	Pelagibacterales	*Candidatus Fonsibacter*
	Rhizobiales	*Bosea*
		*Hyphomicrobium*
		*Methylobacterium*
	Rhodobacterales	*Paracoccus*
	Rickettsiales	*Wolbachia*
	Sphingomonadales	*Novosphingobium*
		*Sphingobium*
		*Sphingomonas*
	Burkholderiales	*Cupriavidus*
		*Lautropia*
		*Paraburkholderia*
		*Ralstonia*
		*Delftia*
		*Collimonas*
		*Duganella*
		*Janthinobacterium*
		*Massilia*
	Neisseriales	*Neisseria*
	Campylobacterales	*Campylobacter*
	Enterobacterales	*Erwinia*
		*Pantoea*
		*Prevotella*
		*Providencia*
		*Serratia*
	Methylococcales	*Methylothermaceae*
	Oceanospirillales	*Marinomonas*
	Pasteurellales	*Haemophilus*
	Pseudomonadales	*Acinetobacter*
		*Moraxella*
		*Pseudomonas*
	Vibrionales	*Vibrio*
	Xanthomonadales	*Rhodanobacter*
		*Stenotrophomonas*

**Figure 1:**
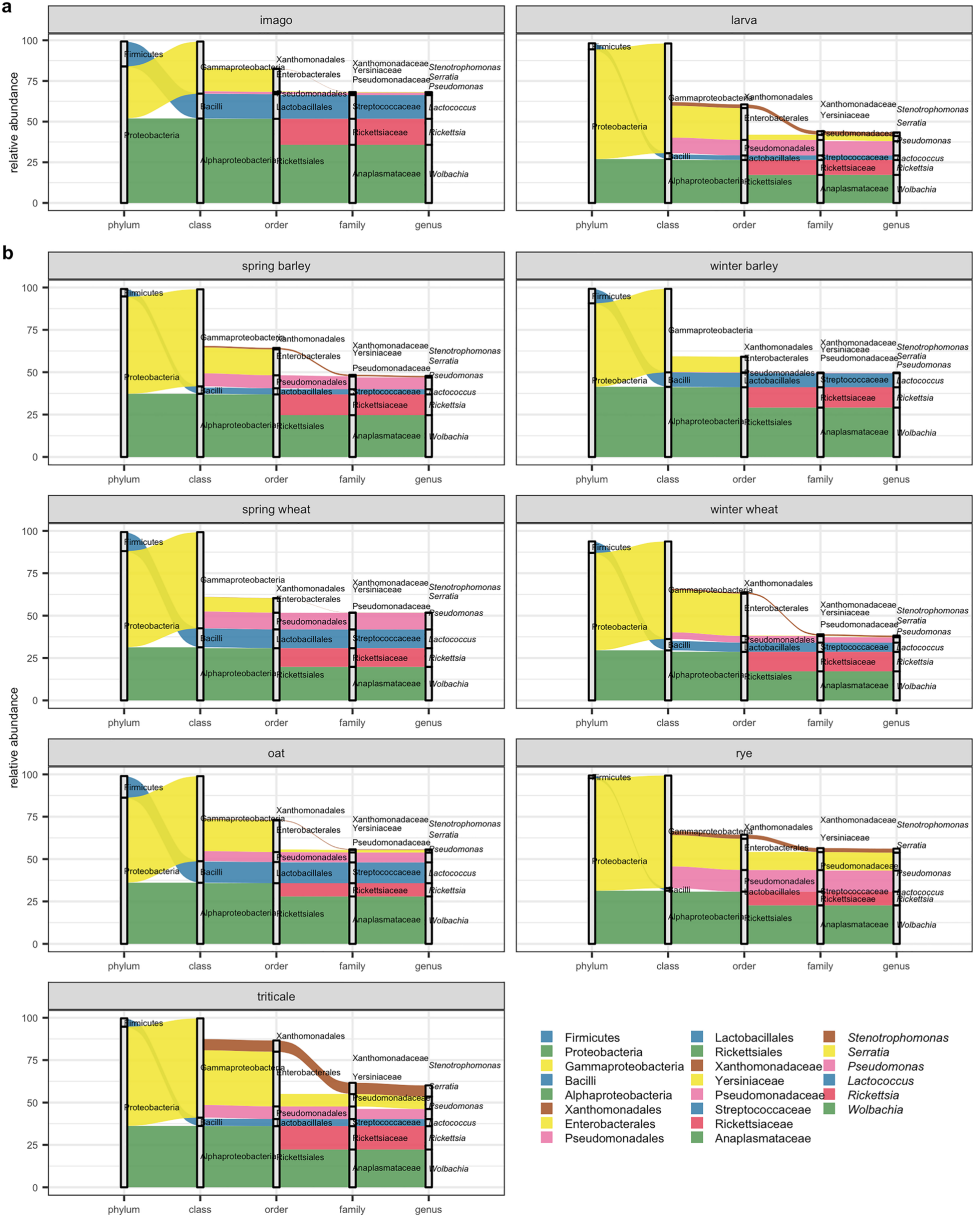
CLB-associated bacteria distribution at phylum, class, order, family, and genus level depending on the variable (**a**) insect’s developmental stage (imago, larva), (**b**) cereal host (spring barley, winter barley, spring wheat, winter wheat, oat, rye, and triticale). The rank taxa with the highest mean value of relative abundance for taxa from Top Biome on each taxonomic level are included in the graph.

After taxonomic evaluation, the relative abundance of the CLB-associated bacteria was determined, including the percentage of unclassified OTUs in the calculation. At each taxonomic level, we calculated the most abundant phyla, meaning the rank taxa with the highest mean value of relative abundance. At the phylum level, the most abundant phyla were Proteobacteria, Firmicutes, Actinobacteria, and Bacteroidetes (Fig. 1a, Supplementary Figure 1S). Proteobacteria comprised on average 91.30% of total reads (94.45% in larval stage and 83.94% in imago) (Fig. 1a, Supplementary Figure 1S). Whereas for Firmicutes it was on average 7.15% (3.66% and 15.31% for larva and imago, respectively), Actinobacteria on average 0.50% (0.47% for larval stage and 0.55% for imago), and Bacteroidetes on average 0.05% (0.03% and 0.09% for larva and imago, respectively) of the total reads. The highest abundance of Firmicutes phyla was observed in insects sampled from oat, spring wheat, winter barley (Fig. 1b, Supplementary Figure 1S).

At the class level, the bacterial communities associated with CLB (both imagoes and larvae) were dominated by Gammaproteobacteria, Alphaproteobacteria, and Bacilli (56.78%, 34.45%, and 7.14%, respectively), which accounted for 98.37% of the total reads. The classes Bacilli and Alphaproteobacteria have higher relative abundances (15.31% and 51.92%) in CLB’s imagoes in comparison to larvae (3.65% and 26.97%, respectively). Opposite results were noted for larvae which were dominated by Gammaproteobacteria (67.40%—larvae, 31.98%—imagoes) (Fig. 1a, Supplementary Figure 1S). The higher abundance of Bacilli class was noted in oat, spring wheat, and winter barley. In turn, a higher percentage of the Gammaproteobacteria was observed in insects feeding on the rye compared to the other cereal crops (Fig. 1b, Supplementary Figure 1S).

At the order level, the CLB bacterial microbiome was dominated by Rickettsiales (34.01%), Enterobacteriales (17.98%), Pseudomonadales (7.08%), and Lactobacillales (6.51%) (Fig. 1a, Supplementary Figure 1S). The orders Lactobacillales (15.16%) and Rickettsiales (51.72%) were relatively more abundant in imago, in comparison to larva (2.80% and 26.42% respectively). In turn, order Pseudomonadales was more abundant in larvae (larval stage—9.53%, imago—1.37%) (Fig. 1a). The order Lactobacillales occurred at significantly higher abundance in insects feeding on the oat, spring wheat, winter barley, in turn, Pseudomonadales were more abundant in insects sampled from rye and spring wheat (Fig. 1b, Supplementary Figure 1S).

At the family level, the most dominant in CLB bacterial population was Anoplasmataceae (22.80%), next to Rickettsiaceae (11.22%), Pseudomonadacea (7.05%), and Streptococcacea (6.34%). The family Anoplasmataceae, Rickettsiaceae, and Streptococacceae were more abundant in adults (35.74%, 15.98%, and 14.61% respectively) than in larvae (17.25%, 9.17%, and 2.80%) (Fig. 1a, Supplementary Figure 1S). A higher abundance of Pseudomonadaceae (9.49%) was noted in larvae (1.34% in imago). The highest abundance of the Pseudomonadaceae family was observed in CLB insects collected from rye, in turn, Streptococcaceae in insects sampled from oat, spring wheat, and winter barley (Fig. 1b, Supplementary Figure 1S).

### Cereal plant host, CLB developmental stage, and insect’s sampling location influence the CLB-associated bacterial community

Based on the preliminary assessment of considered models the hNB GLMM resulted with the best fit based on the R2 coefficient of determination introduced by Nakagawa as well as appropriate estimation of dispersion parameter and fitting considerably high number of zero values in data (see Supplementary Table 5S). That is why it was used for further analysis. The importance of the variables: insect’s developmental stage, the host plants, and insect’s sampling location in shaping the microbiome of CLB at the genus and species level were examined with the usage of the permutational procedure with the Pearson’s statistic used as a goodness of fit measure for the calculations. Results at the genus and species levels indicated that the order of variable importance based on variable-importance measure is as follows: plant hosts, location, and the insect’s developmental stage (Table 2).

**Table 2:** Variable importance based on Pearson’s statistic used as a goodness of fit measure.

Variable	Genus level	Species level
Plant hosts	14.7 * 10^8^	5.98 * 10^8^
Insect’s developmental stage	8.46 * 10^8^	2.34 * 10^8^
Location	11.2 * 10^8^	2.51 * 10^8^

Values represent the penalty for excluding selected variables. The higher penalty the more important variable.

In addition, the possible influence of variables on the bacterial abundance in the microbiome of CLB at the genus and species level was examined using the Wald type II test. The results at the genus level indicated that all three variables are statistically significant at 0.001 level. In turn, test results at the bacterial species level indicated that the host plant and the location from which the insects were collected are statistically significant at 0.001 level, whereas the insect’s developmental stage is statistically significant at 0.01 level. To gain more insight into the differences in CLB bacterial community the data were further analysed using Tukey’s test in multiple comparison procedures. The results suggest that the developmental stage has a significant influence on the CLB microbiome abundance, which was supported by obtaining the significant differences in mean values of bacterial abundances between the imago and larva for genus at 0.001 significance level and species at 0.01 level, respectively. Furthermore, the variable—cereal host may also have a significant influence on the abundance of the CLB microbiome. The differences in mean values of a genus of bacteria abundance were noted as statistically significant for the following pairs of cereals: winter wheat-spring barley, winter wheat-winter barley, spring wheat-winter wheat, winter wheat-rye (p < 0.001), winter wheat-oat (p < 0.01), winter wheat-triticale, rye-triticale (p < 0.05), spring wheat-triticale (p < 0.1) (Supplementary Table 6S). At the bacterial species level for the following pairs of cereals the differences in mean values of bacterial abundances were noted as statistically significant: winter wheat-spring barley, spring wheat-winter wheat, winter wheat-rye (p < 0.001), winter wheat-oat (0.01) rye-triticale (p < 0.05) (Supplementary Table 6S).

At the genus level, all pairs of locations were noted as statistically significant at 0.001 significance level. The differences in mean values of a species were statistically significant at 0.001 level for Zybiszów–Kościelna Wieś pair. Whereas Winna Góra-Kościelna Wieś pair was statistically significant at 0.1 level (Supplementary Table 6S). Further, we mostly focused on two variables: the insect’s developmental stage, and the host plant, because those two factors are relatively more common and relevant for CLB biology. However, the results for variable ‘location’ were placed in the supplementary material (Table 6S).

### Analysis of the biodiversity of CLB-associated bacteria in larva and imago

**Figure 2:**
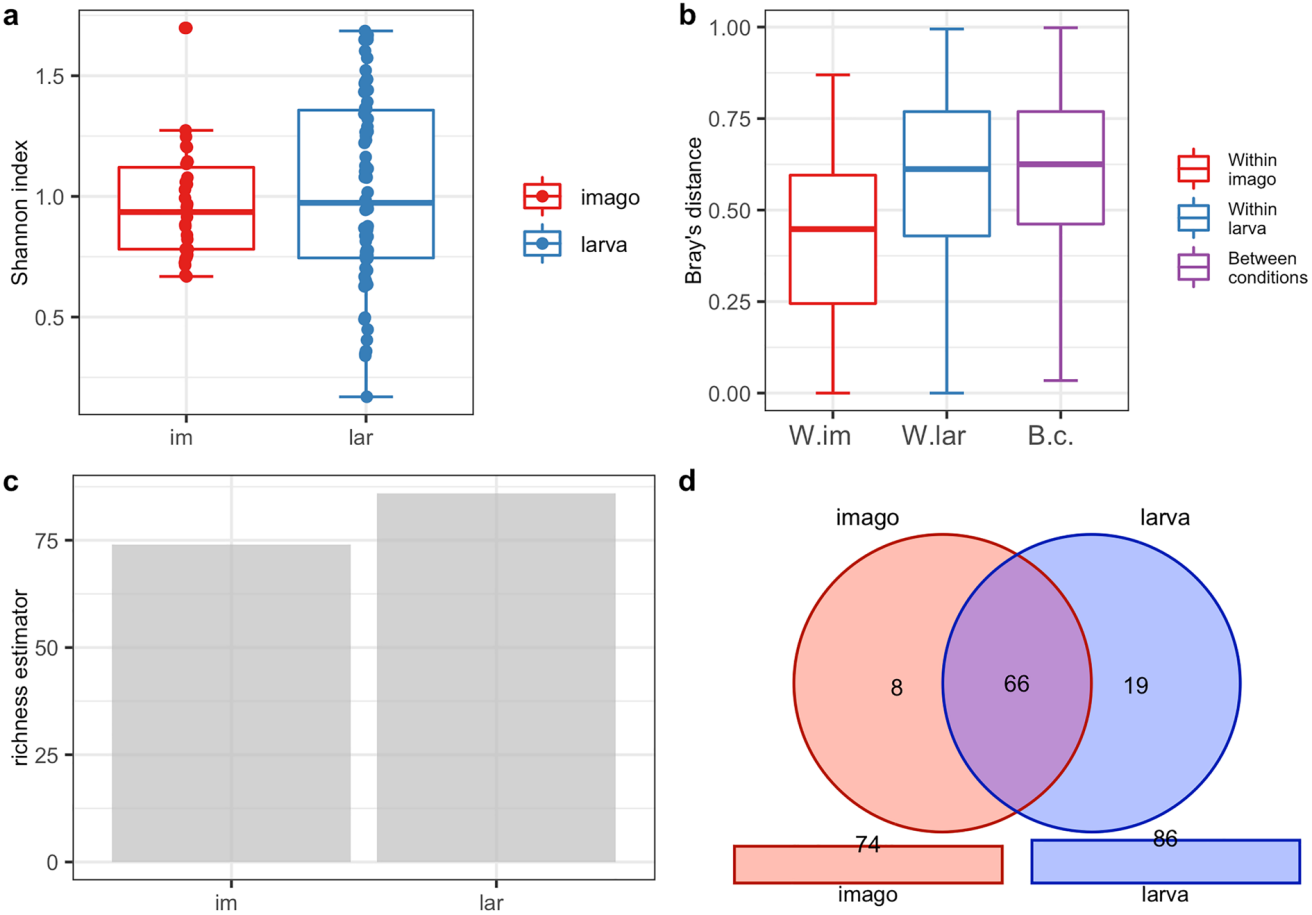
Diversity and richness of CLB bacterial community at the genus level depending on the developmental stage: (**a**) alpha-diversity (Shannon index); (**b**) beta diversity (Jaccard distance); (**c**) level of richness (jacknife coefficient); (**d**) a number of shared genera between imagoes and larvae (Venn’s diagram). im: imago; lar: larva; W.im: within imago; W.lar: within larva; B.c: between conditions.

**Figure 3:**
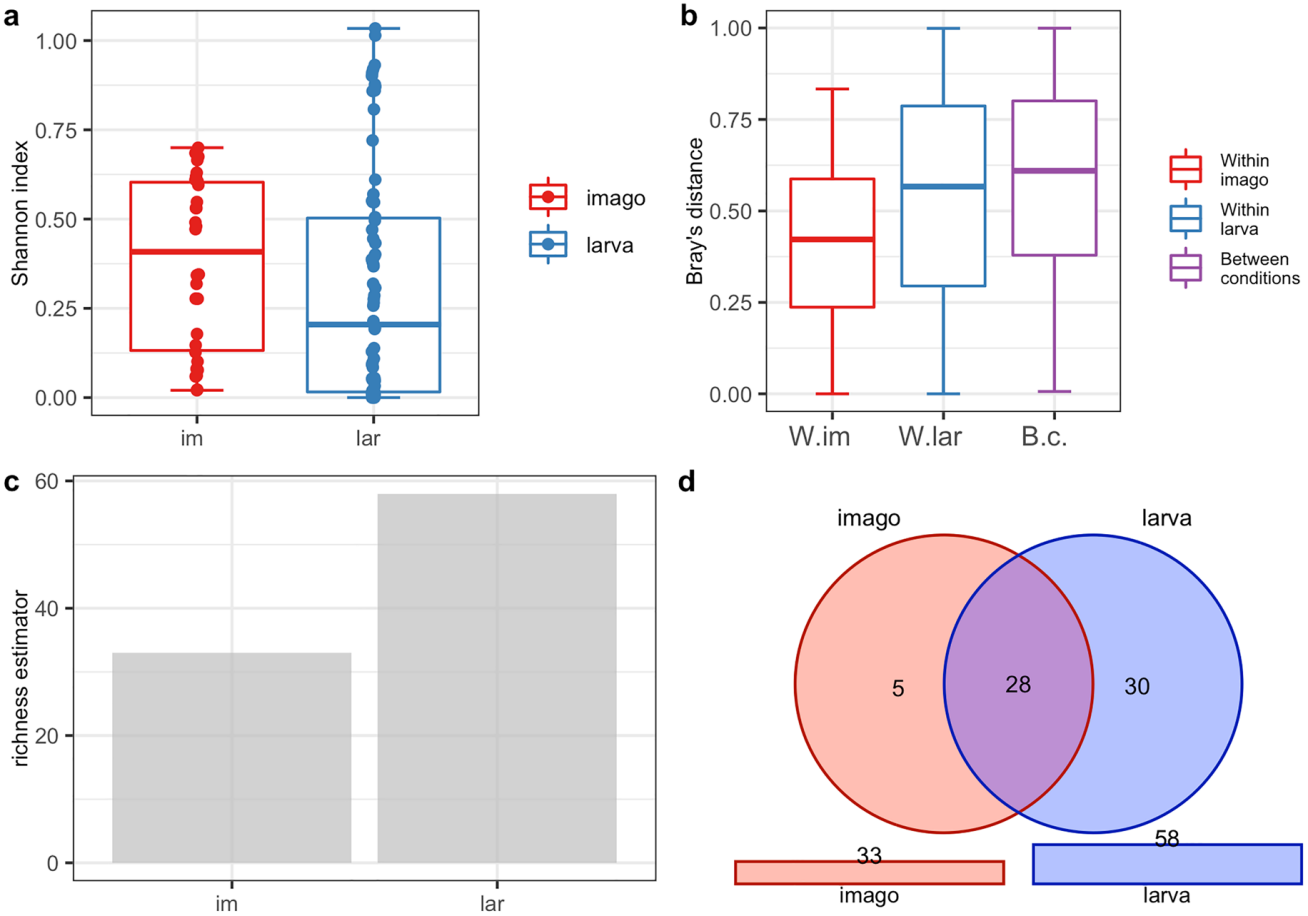
Diversity and richness of CLB bacterial community at the species level depending on the developmental stage: (**a**) alpha-diversity (Shannon index); (**b**) beta diversity (Jaccard distance); (**c**) level of richness (jacknife coefficient); (**d**) a number of shared species between imagoes and larvae (Venn’s diagram). im: imago; lar: larva; W.im: within imago; W.lar: within larva; B.c: between conditions.

The PERANOVA test (the distance between samples measured using the Jaccard, Bray, and Chao similarity method) indicated that differences in the bacterial community between imago and larva were statistically significant for the bacterial genus (p < 0.001) and species level (p < 0.001) (Table 3). The alpha diversity analysis results illustrated with the boxplots for the Shannon index graph (Figs. 2a, 3a) and the beta diversity indices as visualised using the boxplots for Jaccard’s distance (Figs. 2b, 3b) showed that the larvae are characterized by higher bacterial genera (Figs. 2a,b) and species (Fig. 3a,b) diversity in comparison to imagoes with respect to the number of dominant bacterial genera and species as well as the structure of microbiome. The larvae were richer in terms of bacterial genera (Fig. 2c) and species (Fig. 3c), as shown using the jacknife coefficient. The number of identified bacterial genera and species observed in at least one sample for larva (86 and 58, respectively) was higher than for the imago (4 genera, 3 species). Both developmental stages of CLB shared 66 genera (Fig. 2d, Supplementary Table 8S) and 28 species of bacteria (Fig. 3d, Supplementary Table 8S), as illustrated on Venn’s diagram below. The visualization of PCoA based on developmental stages of CLB also revealed that this variable separates data into two groups (Supplementary Figure 2S).

**Table 3:** Alpha and beta diversity statistics regarding CLB’s developmental stage.

Variable: insect’s developmental stage			
Test	p-value		
	Genus level	Species level	
***α*-diversity**			
t-test	0.418		0.252
***β*-diversity** (PERANOVA)			
Jaccard	0.001***	0.001***	
Bray	0.001***	0.001***	
Chao	0.001***	0.001***	

Possible significant codes for p-value: ***0.001, 0.01, 0.05, 0.1, 1

### Analysis of the biodiversity of CLB-associated bacteria in reference to the cereal plant host

**Figure 4:**
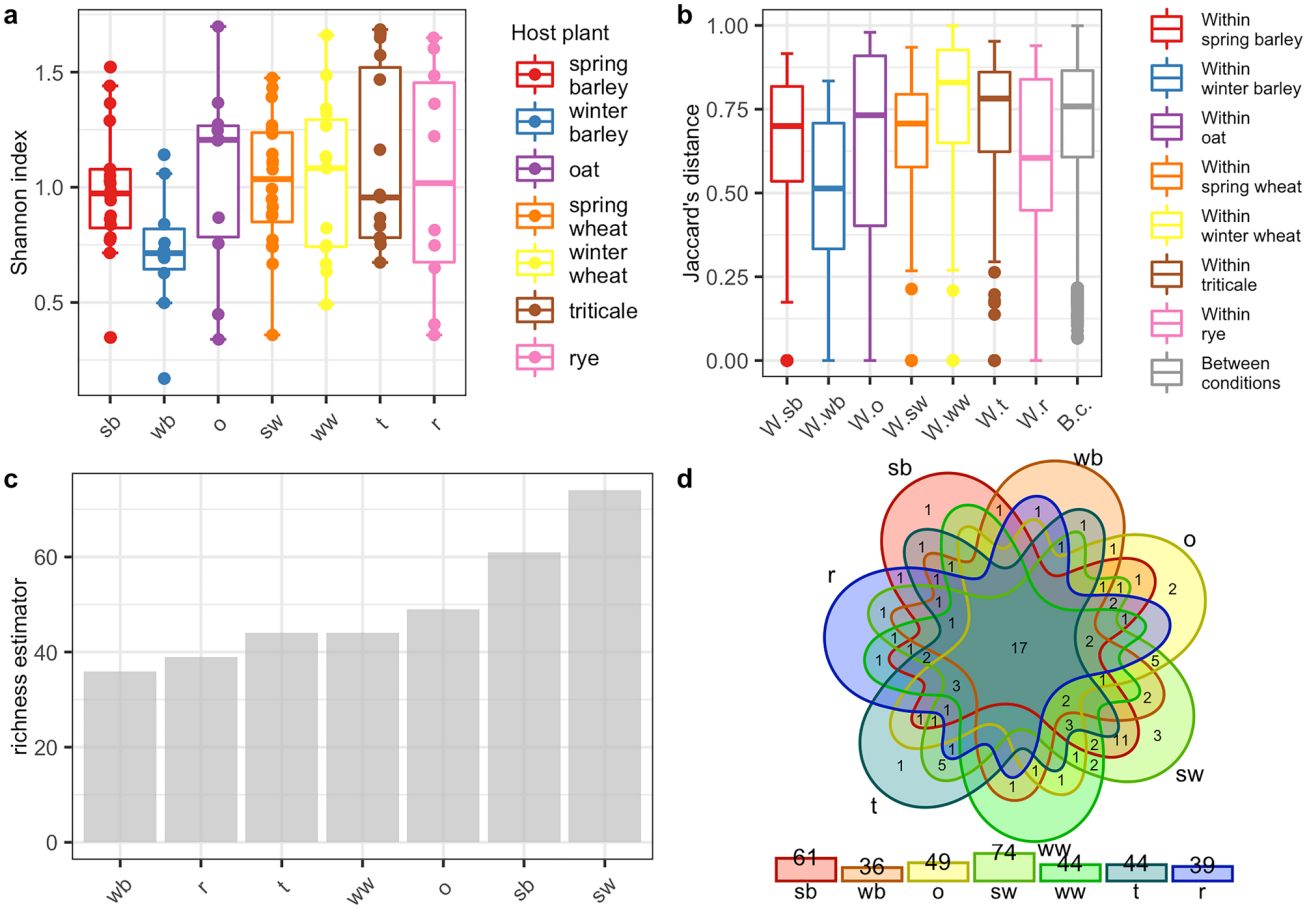
Diversity and richness of bacteria of CLB bacterial community at the genus level depending on the plant host from which insects were collected (**a**) alpha diversity of each group according to the Shannon index, (**b**) beta diversity of each sample according to the Jaccard distance (the vertical lines indicate the range excluding the outliers, the middle lines represent the median value, the boxes represent the upper and lower quartile values, (**c**) the jacknife coefficient determine the level of richness of bacterial community, (**d**) Venn diagram showing the number of shared genera of bacteria between insects collected from various cereal plant hosts observed in at least one sample. sb: spring barley; wb: winter barley; o: oat; sw: spring wheat; ww: winter wheat; t: triticale; r: rye; W.sb: within spring barley; W.wb: within winter barley; W.o: within oat; W.sw: within spring wheat; W.ww: within winter wheat; W.t: within triticale; W.r: within rye; B.c: between conditions.

**Figure 5:**
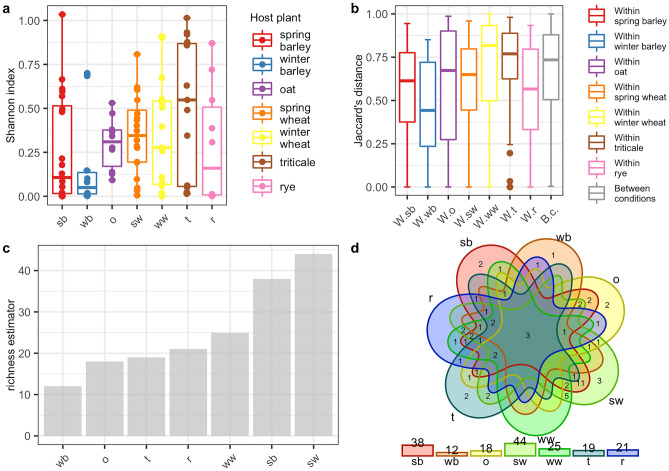
Diversity and bacteria richness of CLB bacterial community for the species level depending on the plant host from which insects were collected (**a**) alpha diversity of each group according to the Shannon index, (**b**) beta diversity of each sample according to the Jaccard distance (the vertical lines indicate the range excluding the outliers, the middle lines represent the median value, the boxes represent the upper and lower quartile values, (**c**) the jacknife coefficient determine the level of richness of bacterial community, (**d**) Venn diagram showing the number of shared bacteria species between insects collected from various cereal plant hosts observed in at least one sample. sb: spring barley; wb: winter barley; o: oat; sw: spring wheat; ww: winter wheat; t: triticale; r: rye; W.sb: within spring barley; W.wb: within winter barley; W.o: within oat; W.sw: within spring wheat; W.ww: within winter wheat; W.t: within triticale; W.r: within rye; B.c: between condition.

The differences in abundance of bacterial genera and species were tested with the Kruskal Wallis test based on the Shannon index. Obtained distributions of Shannon index values suggested that for each cereal host there was a small number of bacterial genera (Fig. 4a) and species (Fig. 5a) that dominated the microbiome. In addition, the number of dominant genera and species between host plants was not statistically significant (Table 4). The variation of microbial compositions between samples was determined by using PERANOVA test based on Jaccard, Bray, and Chao measures of dissimilarity. Obtained results indicated that food sources, in this case, cereal hosts might take part in shaping up the CLB – associated microbiome on both genus and species level (p < 0.05, p < 0.001 respectively) (Table 4). The beta diversity (the Jaccard distance) analysis results showed differences in CLB – associated bacterial community among insects collected from various cereal crops (Figs. 4b, 5b). As shown using the jacknife coefficient, the richness of CLB bacterial genera (Fig. 4c) and species (Fig. 5c) among insects collected from spring barley, spring wheat was significantly higher in comparison to the insects sampled from other cereals. The lowest bacterial richness characterized insects that were feeding on the winter barley and rye (Figs. 4c, 5c). As illustrated on Venn’s diagram insects collected from spring wheat and spring barley were also associated with more genera (Fig. 4d) and species (Fig. 5d) of bacteria in comparison to those sampled from other cereals. Insects feeding on the seven cereal hosts shared 17 genera of bacteria (Fig. 4d, Supplementary Table 7S) and 3 identified bacterial species (Fig. 5d, Supplementary Table 7S). Shared genera and species of bacteria occurred at least once in the group of CLB insects collected from each of the 7 cereal crops. The visualization of PCoA based on the plant host from which insects were collected did not reveal clear differences between considered samples (Supplementary Figure 2S).

**Table 4:** The results of the statistical analysis presented as the p-values of the tests used to characterize the alpha and beta diversity of bacterial genera and species associated with CLB depending on the cereal plant host on which the insects were feeding.

Variable: cereal plant host		
Test	p-value	
	Genus level	Species level
***α*-diversity**		
Kruskal Wallis	0.215	0.147
***β*-diversity** (PERANOVA)		
Jaccard	0.001***	0.001***
Bray	0.001***	0.001***
Chao	0.017*	0.001***

Significant codes for p-value: ***0.001, 0.01, *0.05, 0.1, 1.

### Analysis of the biodiversity of CLB-associated bacteria in reference to the insect’ sampling location

Results of Kruskal Wallis test for alpha diversity (Supplementary Table 7S) as well as distributions of Shannon index values suggested that number of bacterial genera (Fig. 4Sa) and species (Fig. 5Sa) is different between locations. The smallest biodiversity was observed in Kościelna Góra and the highest in Zybiszów. Obtained results of PERANOVA for the variation of microbial compositions between samples (Supplementary Table 7S) as well as distribution of Jaccard’s distance (Supplementary Figures 4Sb, 5Sb) indicated that location might take part in shaping up the CLB-associated microbiome on both genus and species level (p < 0.001, p < 0.05 respectively). The richness of CLB bacteria genera (Fig. 4Sc) and species (Fig. 5Sc) estimated with jacknife coefficient was the highest among insects collected in Winna Góra and the smallest in Kościelna Wieś. As illustrated on Venn’s diagram insects collected at Winna Góra were also associated with more genera (Fig. 4Sd) and species (Fig. 5Sd) of bacteria in comparison to those sampled from other locations. However, visualization of PCoA based on the locations at which insects were collected did not reveal clear differences between considered samples (Supplementary Figure 2S).

### Top biome of CLB associated bacterial genera and species

**Figure 6:**
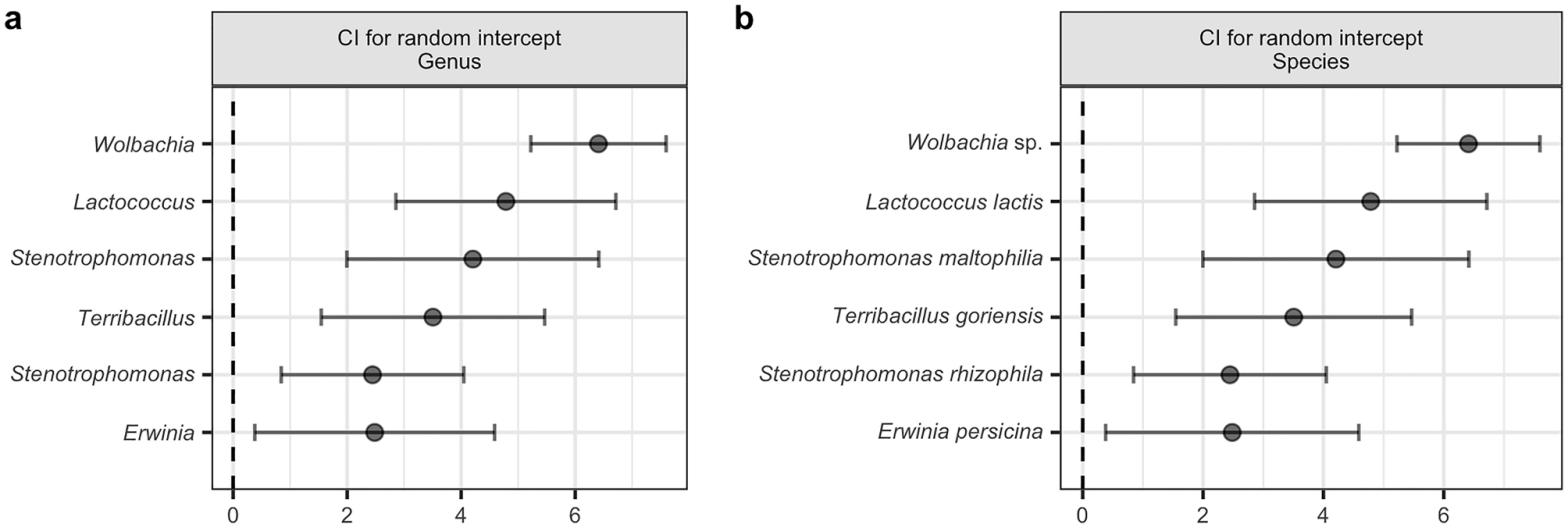
The 99.9% confidence intervals for individual random effects in the hurdle model of the Top Biome consisting of 6 bacterial genera (**a**) and 6 CLB species of bacteria (**b**). The confidence intervals for each taxon are marked. The given level is not significant when the confidence interval crosses zero. On the other hand, if the confidence interval for given taxa is far from zero, it suggests that the microbial abundance of this taxa is significantly different from the overall microbial abundance. Confidence intervals were calculated at 0.001 the significance level.

**Figure 7:**
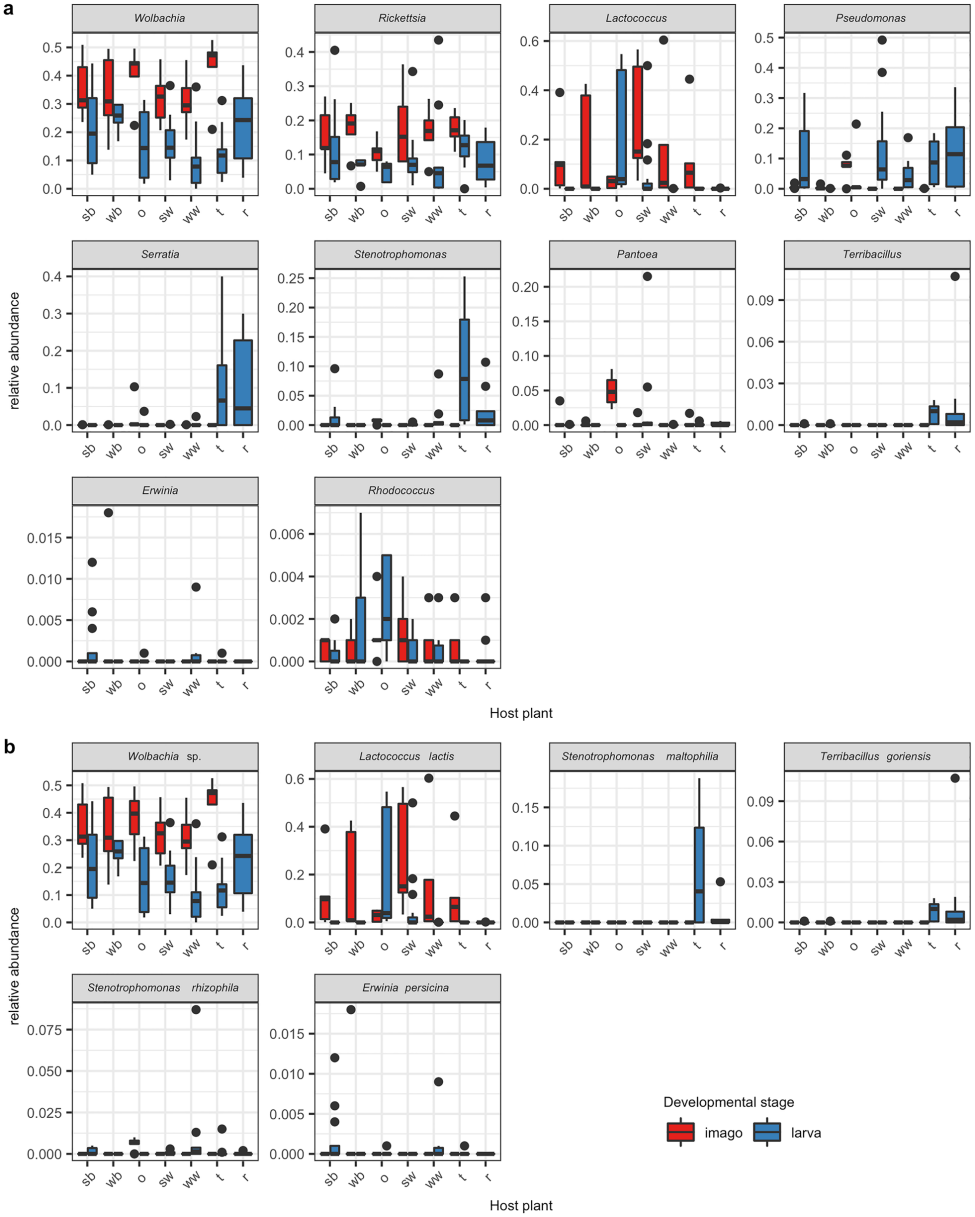
Boxplots visualizing the relative abundance of (**a**) 10 Top Biome genera of bacteria; (**b**) 6 Top Biome identified species of bacteria depending on the variable CLB developmental stage (larva, imago) and cereal plant hosts from which insects were collected. sb: spring barley; wb: winter barley; o: oat; sw: spring wheat; ww: winter wheat; t: triticale; r: rye.

**Figure 8:**
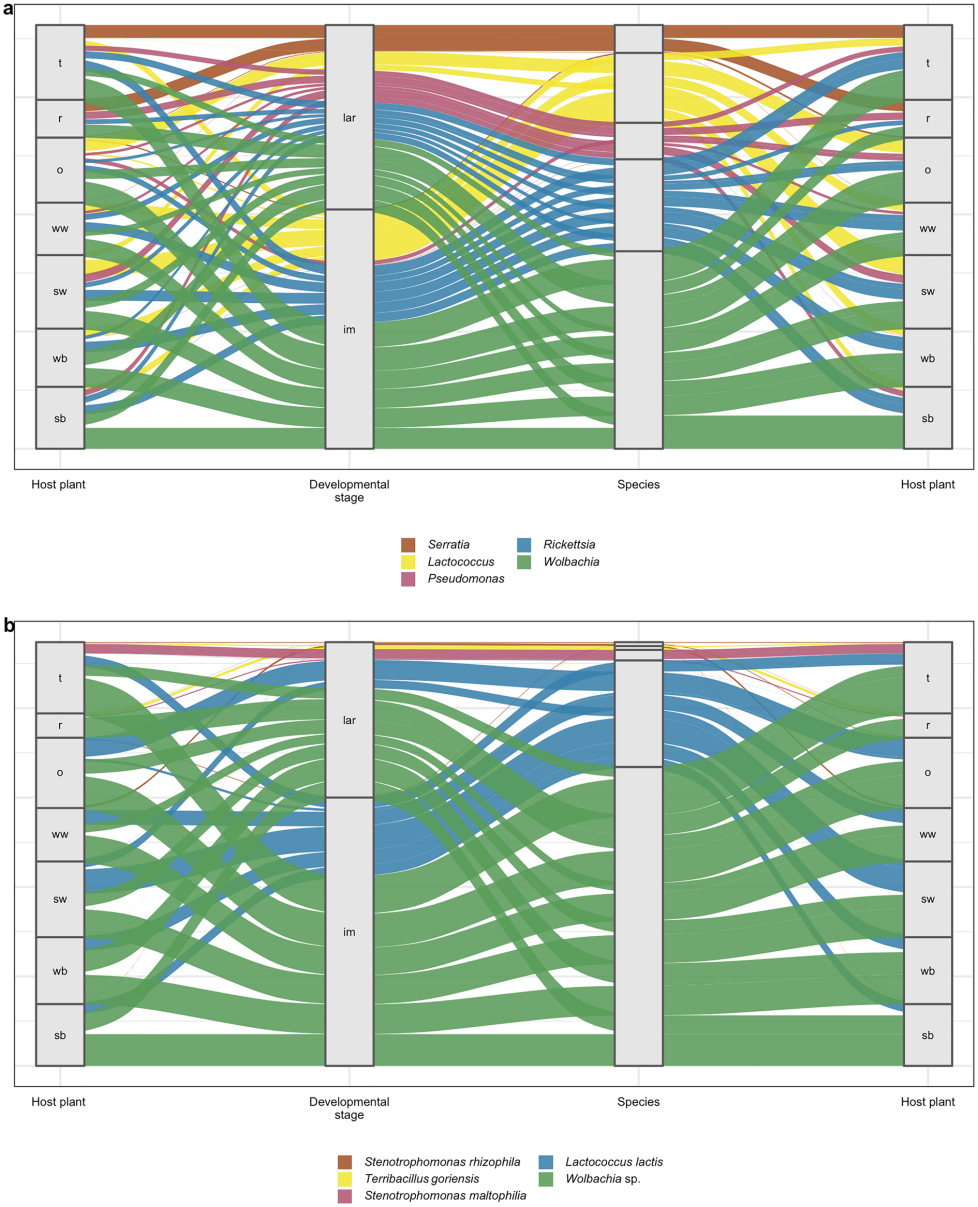
Distribution of 5 the most abundant (**a**) genera and (**b**) species of bacteria of Top Biome depending on the variable: developmental stage (larva, imago) and cereal plant host from which insects were collected. The Sankey diagram has been made on the basis of the percentage values of taxa occurrence excluding samples with zero values. sb: spring barley; wb: winter barley; o: oat; sw: spring wheat; ww: winter wheat; t: triticale; r: rye.

On the basis of the results of the 99.9% confidence intervals for individual random effects in the hurdle model, we cleaved off the most abundant genera that make up the greater part of the CLB microbiome, for which we propose the term Top Biome. For analysed samples of CLB, the Top Biome consists of 10 bacterial genera listed in descending order starting with the most statistically significant: *Wolbachia*, *Rickettsia, Lactococcus, Pseudomonas*, *Serratia*, *Stenotrophomonas*, *Pantoea*, *Terribacillus*, *Erwinia*, *Rhodococcus* (Fig. 6a). The relative abundance of particular genera of bacteria from the Top Biome depends on the insect’s developmental stage and cereal plant host from which insects were sampled. These relations are illustrated using the boxplots (Fig. 7a), the heatmap (Supplementary Figure 3S), the barplot (Supplementary Figure 1S), and the Sankey diagram (Fig. 8a). The most dominant genera in the CLB microbiome were *Wolbachia* (22.80%), *Rickettsia* (11.22%), *Pseudomonas* (6.54%), *Lactococcus* (6.34%), and *Serratia* (2.34%) (Figs. 7a, 8a). Based on differential analysis with DESeq2 method revealed that *Staphylococcus, Vagococcus, Pseudomonas, Stenotrophomonas, Acinetobacter, Moraxella, Sanguibacter, Serratia, Enterococcus, Rickettsia, Lawsonella,* and *Kocuria* were significantly differentiating the microbiomes of larvae and imagoes of CLB at 0.05 level. Whereas, *Acinetobacter, Stenotrophomonas, Streptococcus, Serratia, Moraxella, Lactobacillus, Terribacillus, Pseudomonas, Lawsonella,* and *Pantoea* were significantly differentiating between the microbiomes of insects collected from 7 cereal hosts at 0.05 level. The proportions of *Wolbachia* (35.74%), *Rickettsia* (15.98%), and *Lactococcus* (14.61%) in adults of CLB were significantly higher in comparison to the larval stage (17.25%, 9.17%, and 2.79%, respectively). In turn, larvae contained more *Pseudomonas* (8.76%) and *Serratia* (3.18%) bacteria in comparison to the adults of CLB (1.34%% and 0.37% respectively) (Figs. 7a, 8a). Insects collected from oat and spring wheat were characterized by a higher abundance of genus *Lactococcus* than insects sampled from other crops. Genus *Serratia* was mainly associated with insects feeding on triticale and rye (Fig. 8a).

At the species level, the identified Top Biome consisting of 6 bacterial species (*Wolbachia* sp., *Lactococcus lactis*, *Stenotrophomonas maltophilia*, *Terribacillus goriensis*, *Stenotrophomonas rhizophila*, *Erwinia persicina*) was obtained (Fig. 6b). These species of bacteria, listed in descending order starting with the most statistically significant, are the most abundant and make up the greater part of the identified CLB microbiome. The obtained result suggests that above mentioned bacterial species might be involved in the life processes of an insect. The relative abundance of individual species from the Top Biome is associated with the insect’s developmental stage and cereal plant host on which CLB insects were feeding. These relations are illustrated using the boxplots (Fig. 7b), the heatmap (Supplementary Figure 4S), and the Sankey diagram (Fig. 8b). The following bacteria: *Wolbachia* sp. (22.64%), *L. lactis* (6.33%), *S. maltophilia* (0.69%), are the most abundant in CLB insects. Based on differential analysis with DESeq2 method revealed that *S. maltophilia* and *Sanguibacter keddieii* were significantly differentiating the microbiomes of larvae and imagoes of CLB at 0.05 level. Whereas, *Moraxella osloensis*, *S. maltophilia*, *Lawsonella clevelandensis,* and *Terribacillus goriensis* were significantly differentiating between the microbiomes of insects collected from 7 cereal hosts at 0.05 level.

Higher values of relative abundance of *Wolbachia* sp. (35.29%) and *L. lactis* (14.60%) were observed in imago as compared to larva (17.22% and 2.79% respectively) (Figs. 7b, 8b). *Wolbachia* sp. and *L. lactis* constitute the majority of the identified (at the species level) CLB’s imago microbiome, namely 49.89% of all insect bacterial community, whereas in larva above-mentioned species of bacteria form only 20.01% (Fig. 8b). It is worth noting that the largest share of *L. lactis* was recorded in insects feeding on oat and spring wheat (Figs. 7b, 8b).

### Analysis of the biodiversity of CLB-associated bacteria in reference to the three tested variables

**Figure 9:**
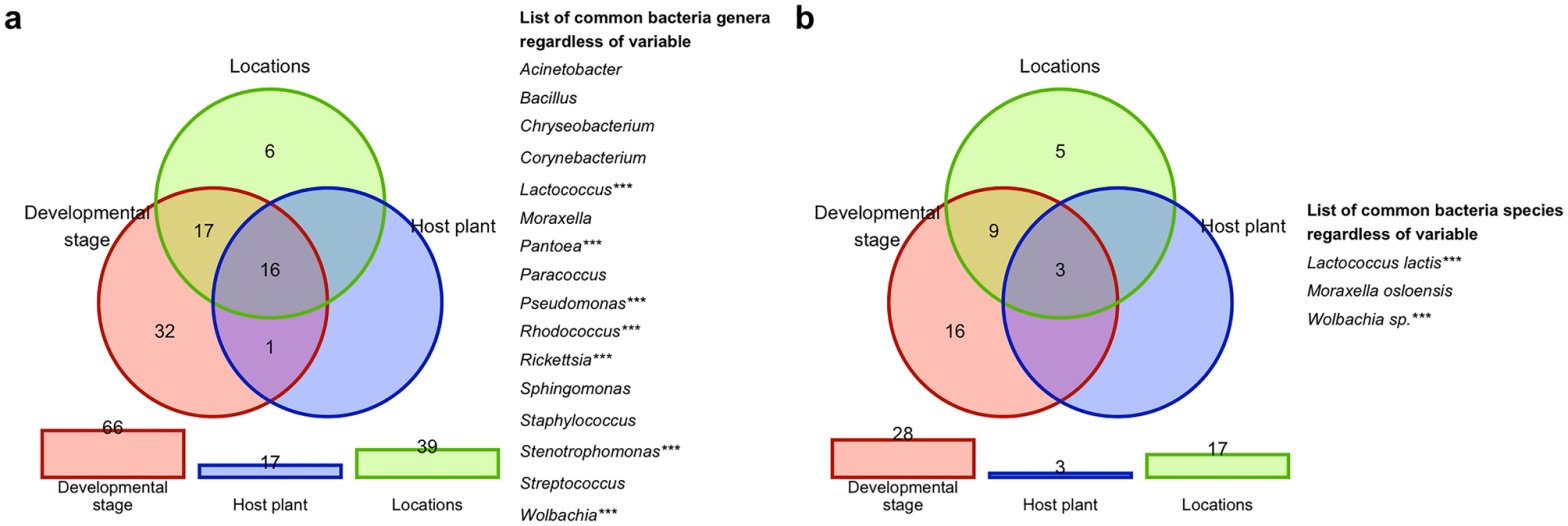
Venn diagram showing the number of common (**a**) genera and (**b**) species of bacteria between insects collected from various cereal plant hosts, locations at different insect’s developmental stages. Bacterial genera marked with asterisks have the highest significance and belong to the Top Biome.

Taking into account only common bacteria genera and species observed in at least one sample for the three variables (developmental stage, plant host, location), it was indicated that the same following 16 genera: *Acinetobacter*, *Bacillus*, *Chryseobacterium*, *Corynebacterium*, *Lactococcus*, *Moraxella*, *Pantoea*, *Paracoccus*, *Pseudomonas*, *Rhodococcus*, *Rickettsia*, *Sphingomonas*, *Staphylococcus*, *Stenotrophomonas*, *Streptococcus*, *Wolbachia* (Fig. 9a) and 3 species of bacteria: *L. lactis*, *Moraxella osloensis*, and *Wolbachia* sp. (Fig. 9b) were observed regardless of the tested variables. Importantly, the shared species of bacteria of CLB collected from seven cereal hosts (with exception of *Serratia*) were the same as the shared microbiome for the tested CLB insects group collected from various locations, hosts, and developmental stages (Supplementary Table 7S).

### Core microbiome—bacteria genera and species shared by the majority of CLB insects regardless of the variables tested

The core microbiome understood as a group of bacterial genera and species shared by the majority of CLB insects regardless of the tested variables (developmental stage, cereal plant host, and location) was specified. It was indicated that at least 90% of tested insects in each group were associated with 2 bacterial genera: *Rickettsia*, *Wolbachia* (Supplementary Figure 7S). In turn, among species of bacteria that we were able to identify, *Wolbachia* sp. was observed in the majority of CLB insects (Supplementary Figure 7S). That is why we suggest that shared above-mentioned genera and species of bacteria may be involved in life processes vital for the CLB.

## Discussion

The CLB bacterial microbiome was determined by using the NGS approach. Our study showed that the bacterial community of CLB is dominated by Proteobacteria and Firmicutes representing 98.34% of all classified sequences (Fig. 1a). This result is supported by a previous study^4^. The predominating classes in CLB insects were Gammaproteobacteria, Alphaproteobacteria, and Bacilli, which accounted for 98.26% of the total reads. At the family level, the most dominant in the CLB population were Anoplasmataceae, Rickettsiaceae, Pseudomonadaceae, and Streptococcaceae (Fig. 1a, Supplementary Figure 1S). The above-mentioned taxa share different percentages in the larva and imago. It is known that the structure and diversity of the insect microbiome are shaped by many factors including the insect’s developmental stage^23^, diet type^26,68^, host taxonomy^24,68^, environment^25^, and social interactions^27^. Our aim was to assess how the developmental stage, the host plant, and insect´ sampling locations shape the structure of the CLB bacterial community and to indicate the core microbiome defined as the genera and species of bacteria associated with the majority of CLB insects, regardless of the variables analysed.

According to our results, the bacterial community of CLB is shaped by diet, the insect developmental stage, and locations (Table 2). It is known that food source influences insect gut microbial community in response to dietary changes through the induction of enzymes and affects bacterial profile^26,69^. This result is supported by another report^70^ where it was indicated that the community structure of several facultative microbes associated with the Asian rice gall midge (*Orseolia oryzae*, Cecidomyiidae) depended largely on the diet and also on developmental stage^70^. This result was also supported by other research^71^, where it was noted that the structure of the bacterial community of the *Helicoverpa zea* (Noctuidae) depended on the host plant.

Interestingly in our study, it was indicated that insects feeding on the spring wheat and spring barley were associated with a much larger number of bacterial genera and species in comparison to insects sampled from other cereals (Figs. 4, 5). This observation is in congruence with other studies that showed that insect gut microbiota changes within season^72^. In addition, the concentration of secondary compounds of leaves may affect the bacterial community^73^. That is why the differences in concentration of secondary compounds and the bacterial composition of cereal leaves, on which insects were feeding, may explain the differences in bacterial biodiversity observed between CLB insects’ samples collected in this study from various cereals.

The composition of the insect microbiome does not accurately reflect the microbiome of the plants they feed on, as statistically significant differences in biodiversity and number of species and genera of bacteria in the CLB microbiome between larva and imago were noted (Figs. 2, 3). In the insect gut, the filtration of certain taxa of bacteria from a wider environmental pool of bacteria takes place^74^. It is known that the bacterial structure of insects microbiome is shaped by a number of insect-host-related factors^71^ including physical and physiological conditions in the insect gut such as pH, redox potential, substrates availability^22^. Secondly, the composition of bacterial taxa can depend on the insect’s immune system^75^. Usually, insects initiate an immune response against the pathogenic bacteria (including phagocytosis, melanization, encapsulation, nodulation, lysis, RNAi-mediated virus destruction, autophagy, and apoptosis)^76^ but can selectively maintain the beneficial microbes^74,77^. Thirdly, various bacteria species can live together in insect hosts without any special relationship between them, but there are also species whose abundance depends on the presence and prevalence of other bacterial species^78^. Bacteria live within an insect body and share limited resources. Competition amongst the co-residing microbes is inevitable when the food source is limited. The competition among taxa may also affect the structure of the insect's bacterial community.

The most abundant identified species of bacteria in the CLB population, referred to by us as the Top Biome, are as follows: *Wolbachia* sp., *Lactococcus lactis*, *Stenotrophomonas maltophilia*, *Terribacillus goriensis*, *Stenotrophomonas rhizophila*, *Erwinia persicina* (Fig. 7b)*.* Interestingly, higher values of relative abundance of *Wolbachia* sp., and *L. lactis* were noted in imago and constituted a large part (49.89%) of the CLB’s microbiome, whereas in larva above-mentioned species of bacteria constituted only 20.01% of insect-associated bacteria (Figs. 7b, 8b). In contrast, the larvae bacterial community was characterized by a higher bacterial diversity (Figs. 2, 3). It is important, however, that obtained taxonomy result has some limitations resulting from the applied Kraken2 confidence threshold of 0.05, which is being used as the lowest limit in current NGS experiments^79,80^. The analysis performed at the 0.04 and 0.03 (data not shown) has revealed the same statistical significance of the tested variables, but with higher bacterial diversity of both imago and larva.

The differences in bacterial structure between the larva and imago may be due to their feeding habits. It is known, that CLB larvae are less mobile than adult beetles and their feeding intensity is high, which is why larvae are considered more harmful than imagoes and cause considerable damage to plants. In turn, adult beetles after supplementary feeding are mainly focused on reproduction (insects die after laying eggs). These differences in feeding habits may provide differential binding and colonization affinities for microbiota resulting in a greater diversity or abundances of microbial taxa. The dominance of the genus *Wolbachia* and *Rickettsia* in imago may be associated with insect reproduction and maturation of their sexual organs since these species are known as reproductive manipulators^81^ and may affect multiple aspects of insect host biology. *Wolbachia* can be mutualistic and increase the insect host lifespan, fecundity, providing vitamins and nutrients, defense against viruses and parasites^82^ or can be parasitic through feminization of genetic males, male-killing, parthenogenesis, and cytoplasmic incompatibility. It was estimated that *Wolbachia* infects more than 65% of all insect species^81,83,84^, including numerous genera of beetles such as *Ips*, *Xyleborus*, *Xylosandrus*, *Coccotrypes*^82^. Many species belonging to the above-mentioned genera were characterized by high infection rates by these bacterial species (70–100%)^82^.

*L. lactis* is also a dominant bacteria species in imago (Figs. 7b, 8b). This species belongs to the Firmicutes (Fig. 1a, Supplementary material 1S) which is known to contribute to the decomposition of complex carbohydrates, fatty acids, or polysaccharides in the insect gut^85–87^. It might also contribute to the improvement of nutrient availability^88^. Probably these species of bacteria play an important role in the digestive process of the CLB insects as well.

Genus *Pantoea* was noted for both larva and imago, but its abundance is quite low (0.46%—larva, 1.09%—imago). Some species of *Pantoea* are known to participate in the degradation, utilization of different types of plant materials^14,89^, thus it can be hypothesized that this genus plays also an important role in CLB digestion of plant material. It is worth mentioning that part of the CLB insects was associated with species of bacteria known as plant pathogens, such as *Erwinia persicina*. Recently, we reported that CLBs were associated with four strains of *P. ananatis*, which were able to fully develop disease symptoms on wheat plants. *P. ananatis* was also identified in numerous samples of CLB insects in this study when Kraken2 results were filtered using a slightly lower confidence threshold (0.04).

CLB larvae were associated with the higher relative abundance of *Pseudomonas* genus as compared to imago (Fig. 1a, Supplementary material 1S). *Pseudomonas* genus can be involved in the digestion of insect host’s food and plant secondary metabolites, which may increase the availability of nutrients^85,90,91^. For instance, one of the most devastating pests of coffee, the coffee berry borer (*Hypothenemus hampei*, Curculionidae), carries a *Pseudomonas* bacterium in the gut that degrades caffeine and allows insect survival on coffee fruits^92^. Some species of *Pseudomonas* are involved in the development of pesticide resistance in their insect host, as was reported for *Spodoptera frugiperda*^34^.

## Conclusions

Our analysis showed that *Wolbachia* and *Rickettsia* constitute the core microbiome of CLB. Those bacteria were present in almost all of the analysed CLB insects, regardless of the variables tested. That is why we suggest that these bacterial genera may be essential for the CLB and might be involved in life processes vital for the insect. Presented results extend the knowledge on the microbiome composition and diversity of the serious cereal pest—CLB. We indicated that diet, the developmental stage, and locations from which insects were collected are shaping the bacterial community structure of CLB. Targeting the aforementioned CLB-associated species of bacteria can be the basis for developing integrated, environmentally friendly, and sustainable pest-management strategies to limit CLB damages.

## ﻿Supplementary Information

Supplementary Information.

## Data Availability

All data generated or analyzed in this study are included in this article and the Supplementary Information Files.
