# Supplementary material for: The structure of the cereal leaf beetle (Oulema melanopus) microbiome depends on the insect’s developmental stage, host plant, and origin
Source: Sci Rep. 2021 Oct 14;11:20496. doi: 10.1038/s41598-021-99411-9 (PMC8516949; doi:10.1038/s41598-021-99411-9)
Supplement: Supplementary file 1 — Supplementary Information. [file 41598_2021_99411_MOESM1_ESM.pdf]

Supplementary material

The structure of the cereal leaf beetle (*Oulema melanopus*) microbiome depends on the insect`s developmental stage, host plant, and origin

Beata Wielkopolan (ORCID 0000-0001-5734-9148) [1], Krzysztof Krawczyk (ORCID 0000-0002-6279-4921) [2], Alicja Szabelska-Beręsewicz (ORCID 0000-0002-1806-0891) [3], Aleksandra Obrępalska-Stęplowska (ORCID 0000-0002-0314-8110) [2]

1) Department of Monitoring and Signaling of Agrophages, Institute of Plant Protection – National Research Institute, 20 Węgorka St, 60-318 Poznań, Poland
2) Department of Molecular Biology and Biotechnology, Institute of Plant Protection – National Research Institute, 20 Węgorka St, 60-318 Poznań, Poland
3) Department of Mathematical and Statistical Methods, Poznań University of Life Sciences, 28 Wojska Polskiego St, 60-624 Poznań, Poland

Table 1S. The list of CLB insect samples, indicating the developmental stage, the host plant and the locations from which insects were collected

**Sample numer**					**Developmental stage**	**Host plant**	**Locations**
1	2	3	4	5	larva	triticale	Kościelna Wieś
6	7	8	9	10	larva	winter wheat	Winna Góra
11	12	13	14	15	larva	spring wheat	Winna Góra
16	17	18	19	20	larva	winter barley	Kościelna Wieś
21	22	23	24	25	larva	rye	Kościelna Wieś
26	27	28	29	30	larva	oat	Winna Góra
31	32	33	34	35	larva	rye	Zybiszów
36	37	38	39	40	larva	spring wheat	Zybiszów
41	42	43	44	45	larva	triticale	Zybiszów
46	47	48	49	50	larva	winter wheat	Zybiszów
51	52	53	54	55	larva	spring barley	Zybiszów
56	57	58	59	60	larva	spring barley	Kościelna Wieś
61	62	63	64	65	imago	spring wheat	Winna Góra
66	67	68	69	70	imago	triticale	Winna Góra
71	72	73	74	75	imago	spring barley	Winna Góra
76	77	78	79	80	imago	winter wheat	Winna Góra

81	82	83	84	85	imago	winter barley	Winna Góra
86	87	88	89	90	imago	oat	Winna Góra
91	92	93	94	95	larva	spring barley	Zybiszów
96	97	98	99	100	larva	spring barley	Winna Góra

Table 2S. The number of raw reads and reads obtained after trimming, filtering. The resulting sequences were used for taxonomic classification. The taxonomic placement was performed using the lowest common ancestor (LCA) algorithm Kraken 2 implemented in OmicsBox software (v. 1.4.11). The percentage of sequences classified to the given taxonomic level is given

Percentage of Classification									
**Sample**	**Raw reads**	**Reads obtained after trimming, filtering**	**Superkingdom**	**Phylum**	**Class**	**Order**	**Family**	**Genus**	**Species**
1	46734	14813	100.00%	100.00%	100.00%	61.27%	21.81%	21.73%	14.45%
2	52926	18502	100.00%	99.98%	99.98%	94.97%	17.14%	16.31%	2.83%
3	67838	26375	99.98%	99.95%	99.86%	99.22%	53.11%	40.44%	31.66%
4	79644	25891	100.00%	100.00%	100.00%	90.94%	39.01%	38.63%	23.67%
5	55416	17597	100.00%	100.00%	100.00%	90.23%	37.82%	31.83%	8.12%
6	77086	25303	100.00%	100.00%	99.99%	96.66%	25.34%	23.38%	11.74%
7	97246	33877	100.00%	99.95%	99.95%	43.82%	43.79%	42.87%	35.99%
8	89720	30245	99.99%	99.96%	99.95%	34.10%	34.02%	33.88%	9.07%
9	28468	9347	100.00%	100.00%	100.00%	35.86%	35.40%	30.50%	23.79%
10	73136	27658	99.76%	99.70%	99.70%	74.33%	73.64%	70.77%	7.06%
11	91066	36261	100.00%	99.98%	99.94%	66.88%	65.26%	64.98%	16.78%
12	92072	31365	99.98%	99.95%	99.93%	72.03%	24.27%	23.91%	13.23%
13	111088	39573	99.97%	99.97%	99.97%	15.66%	14.93%	14.69%	4.52%
14	90640	32261	99.99%	99.99%	99.99%	57.56%	51.25%	50.91%	36.64%
15	132730	46180	99.98%	99.98%	99.98%	60.10%	49.98%	48.52%	26.83%
16	104810	37608	100.00%	99.95%	99.95%	35.32%	34.85%	35.08%	26.50%
17	68644	23401	99.99%	99.98%	99.98%	38.22%	36.89%	36.89%	29.78%
18	107388	41244	99.98%	99.91%	99.89%	30.77%	30.76%	30.74%	29.82%
19	127488	44012	99.99%	99.77%	99.74%	24.89%	24.81%	24.44%	16.91%
20	145918	52289	99.99%	99.58%	99.57%	34.08%	34.08%	32.76%	23.55%
21	110920	41003	99.98%	99.84%	99.83%	49.51%	49.38%	48.87%	43.72%
22	106436	39791	100.00%	99.35%	99.31%	53.31%	50.43%	49.66%	33.36%
23	105606	42423	100.00%	99.87%	99.86%	54.14%	53.30%	52.55%	38.00%
24	88118	31415	99.99%	98.86%	98.84%	49.61%	31.49%	31.08%	28.39%
25	108500	37993	99.98%	99.88%	99.87%	45.29%	31.30%	30.60%	24.77%
26	88904	28769	100.00%	99.92%	99.92%	81.94%	50.62%	49.65%	16.42%
27	126366	45153	99.99%	99.99%	99.98%	82.06%	54.42%	54.34%	52.12%
28	100300	36328	100.00%	100.00%	99.98%	79.20%	59.36%	58.89%	56.41%
29	106300	37634	100.00%	99.96%	99.96%	37.26%	36.85%	36.21%	27.63%
30	151150	52953	99.99%	99.96%	99.93%	59.96%	43.65%	42.93%	35.36%
31	113570	37563	100.00%	99.95%	99.95%	79.65%	55.27%	52.42%	20.78%
32	131372	44374	100.00%	98.68%	98.66%	81.60%	80.44%	77.72%	19.27%

33	101802	31344	99.97%	99.95%	99.95%	87.62%	82.20%	80.10%	4.80%
34	133716	44426	99.99%	99.83%	99.81%	88.87%	83.66%	81.90%	31.30%
35	122468	42483	100.00%	99.21%	99.20%	80.32%	79.41%	78.31%	7.70%
36	87952	31300	99.96%	97.35%	97.26%	59.47%	51.18%	45.54%	27.09%
37	125272	43008	99.99%	98.37%	98.37%	57.63%	53.69%	50.34%	11.14%
38	146810	34554	100.00%	99.76%	99.76%	87.56%	78.60%	75.86%	12.51%
39	134456	52042	99.93%	99.14%	99.11%	61.75%	58.87%	56.69%	21.55%
40	79638	27914	99.99%	99.01%	98.96%	55.16%	54.14%	52.13%	29.09%
41	92782	25784	100.00%	100.00%	99.99%	98.38%	93.55%	88.03%	13.26%
42	93892	28640	99.99%	99.68%	99.66%	89.56%	76.98%	71.63%	21.28%
43	89906	28386	100.00%	99.75%	99.69%	90.00%	86.16%	80.70%	32.42%
44	110190	31417	100.00%	99.96%	99.95%	95.85%	87.92%	82.53%	21.43%
45	93520	28429	99.99%	99.79%	99.72%	94.90%	83.80%	79.28%	27.77%
46	105864	40736	99.99%	98.91%	98.90%	87.96%	4.00%	3.10%	1.55%
47	116756	40435	99.97%	84.84%	84.82%	13.34%	11.63%	5.90%	3.88%
48	76384	27393	100.00%	99.74%	99.72%	94.64%	30.03%	26.38%	11.08%
49	144094	53774	100.00%	87.62%	87.58%	75.63%	45.34%	26.30%	10.13%
50	111422	39463	100.00%	54.64%	54.62%	52.52%	24.98%	13.26%	6.03%
51	149888	52700	100.00%	94.93%	94.91%	64.37%	63.65%	61.86%	20.10%
52	60956	22492	100.00%	99.90%	99.90%	96.47%	14.97%	14.58%	6.20%
53	101800	35618	99.99%	99.68%	99.67%	56.16%	48.36%	47.39%	29.33%
54	118660	44706	100.00%	99.80%	99.77%	48.76%	48.63%	47.91%	42.95%
55	116376	46209	100.00%	99.46%	99.44%	63.11%	63.02%	62.25%	35.56%
56	81620	28750	100.00%	99.94%	99.94%	57.83%	56.02%	55.17%	36.95%
57	74304	24965	99.99%	99.72%	99.61%	50.95%	44.82%	40.94%	13.92%
58	94392	35121	100.00%	99.76%	99.76%	63.63%	62.20%	60.81%	45.02%
59	101362	33204	100.00%	99.98%	99.98%	85.26%	65.66%	64.89%	6.96%
60	97394	36763	100.00%	99.94%	99.92%	67.90%	66.24%	65.30%	28.60%
61	125932	47242	100.00%	99.97%	99.97%	90.19%	90.18%	90.04%	81.90%
62	104926	39830	100.00%	99.90%	99.90%	90.36%	78.69%	78.38%	70.32%
63	112574	41384	100.00%	99.78%	99.78%	73.94%	73.78%	73.14%	36.17%
64	79270	29626	100.00%	99.82%	99.80%	81.63%	77.99%	77.53%	51.52%
65	104332	39064	100.00%	99.75%	99.69%	74.84%	74.70%	74.29%	58.49%
66	92304	34144	100.00%	99.80%	99.79%	82.54%	67.74%	67.14%	47.78%
67	96544	34348	99.99%	99.94%	99.94%	94.75%	76.58%	76.46%	65.58%
68	114486	41271	99.99%	99.93%	99.92%	75.51%	74.93%	74.49%	53.37%
69	916772	34324	99.99%	99.75%	99.72%	72.85%	72.64%	72.24%	48.35%
70	63140	9220	100.00%	100.00%	100.00%	83.17%	74.46%	74.36%	59.08%
71	99056	37236	99.98%	99.75%	99.73%	79.36%	77.09%	76.68%	52.76%
72	63392	21465	100.00%	100.00%	100.00%	97.62%	51.78%	51.71%	39.59%
73	116464	43080	99.99%	99.90%	99.90%	79.77%	79.25%	78.65%	51.02%
74	109082	38139	99.99%	99.98%	99.98%	92.04%	71.17%	71.11%	62.73%
75	100270	11805	100.00%	100.00%	100.00%	94.96%	56.89%	45.30%	32.82%
76	74610	26834	99.99%	99.93%	99.91%	79.21%	63.07%	62.72%	36.19%
77	111294	39520	100.00%	99.96%	99.94%	78.73%	66.43%	66.13%	46.01%
78	93164	31217	99.99%	99.99%	99.99%	93.89%	46.31%	46.25%	32.01%
79	95698	35357	100.00%	99.91%	99.91%	83.05%	83.04%	82.86%	77.69%
80	98856	37956	99.99%	99.85%	99.83%	63.78%	63.61%	62.60%	45.05%
81	86824	29389	100.00%	99.91%	99.91%	80.47%	66.27%	66.08%	46.28%
82	133378	49576	100.00%	99.58%	99.58%	78.79%	76.57%	76.29%	50.54%

83	137140	51296	100.00%	99.92%	99.91%	88.56%	86.59%	86.22%	68.88%
84	104606	36850	100.00%	100.00%	100.00%	93.54%	49.11%	48.92%	26.80%
85	124648	44066	100.00%	99.98%	99.98%	96.16%	70.86%	70.65%	58.26%
86	222902	91134	100.00%	99.92%	99.91%	89.44%	84.70%	83.79%	46.85%
87	219950	91752	100.00%	99.88%	99.88%	75.26%	73.82%	71.55%	34.14%
88	151742	62435	100.00%	99.94%	99.93%	82.99%	79.96%	79.55%	50.71%
89	182718	77173	100.00%	99.87%	99.83%	67.61%	66.27%	65.07%	48.30%
90	225654	93909	100.00%	99.99%	99.99%	91.19%	51.27%	51.10%	27.30%
91	166654	67968	100.00%	99.89%	99.88%	35.70%	34.29%	33.65%	26.79%
92	123840	49692	100.00%	100.00%	100.00%	92.82%	92.39%	92.35%	56.19%
93	101904	41196	100.00%	99.44%	99.42%	22.97%	22.97%	22.57%	16.86%
94	95928	38080	100.00%	99.85%	99.85%	17.58%	17.56%	17.42%	15.62%
95	171630	65494	100.00%	99.86%	99.85%	53.87%	36.97%	32.36%	14.80%
96	184580	76305	99.99%	99.88%	99.88%	42.22%	38.20%	35.03%	12.45%
97	123634	46048	100.00%	99.46%	99.45%	12.05%	11.22%	10.25%	5.94%
98	163686	60601	100.00%	100.00%	100.00%	93.06%	44.77%	41.67%	7.58%
99	137828	55032	100.00%	99.98%	99.98%	47.59%	29.94%	23.67%	14.06%
100	130948	51432	100.00%	99.70%	99.70%	30.75%	30.39%	29.27%	21.09%

Table 3S. Weather conditions at locations during the time when samples were collected

Date	Location	Average monthly temperature (ºC)	Maximum temperature (ºC)	Minimum temperature (ºC)	Total rainfall (mm)
May 2018	Winna Góra	17.8	24.1	10.3	15.1
	Zybiszów	15.98	22.72	9.23	30.86
	Kościelna Wieś	17.1	23.03	11.17	51.6
June 2018	Winna Góra	19.3	25.8	12.9	24.12
	Zybiszów	19.40	24.74	12.02	17.13
	Kościelna Wieś	18.83	24.32	13.35	55
May 2019	Winna Góra	11.8	17.4	5.8	63.25
	Zybiszów	11.32	18.1	4.53	69.9
	Kościelna Wieś	12.25	17.09	7.42	51.4
June 2019	Winna Góra	22.9	30.1	15.1	4.57
	Zybiszów	21.98	29.61	14.17	21.4
	Kościelna Wieś	22.14	28.72	15.56	29.1

Table 4S. Lists of classified bacteria taxa at phylum, class, order, family, genus, and species taxonomic units. The taxonomic placement was performed using the lowest common ancestor (LCA) algorithm Kraken 2 implemented in OmicsBox software (v. 1.4.11), using 0.05 confidence threshold

Phylum	Class	Order	Family	Genus	Species
Acidobacteria	Acidobacteriia	Bryobacterales	Solibacteraceae	*Candidatus* Solibacter	*Candidatus* Solibacter usitatus
Actinobacteria	Acidimicrobiia	Acidimicrobiales	Ilumatobacteraceae	*Ilumatobacter*	*Ilumatobacter coccineus*
	Actinomycetia	Actinomycetales	Actinomycetaceae	*Actinomyces*	not classified
		Corynebacteriales	Corynebacteriaceae	*Corynebacterium*	*Corynebacterium imitans*
					Corynebacterium segmentosum
					Corynebacterium ureicelerivorans
			Lawsonellaceae	*Lawsonella*	*Lawsonella clevelandensis*
			Nocardiaceae	*Rhodococcus*	not classified
		Micrococcales	Brevibacteriaceae	*Brevibacterium*	not classified
			Microbacteriaceae	*Frondihabitans*	*Frondihabitans* sp.
			Microbacteriaceae	*Microbacterium*	not classified
			Micrococcaceae	*Arthrobacter*	not classified
			Micrococcaceae	*Kocuria*	not classified
			Sanguibacteraceae	*Sanguibacter*	*Sanguibacter keddieii*
		Propionibacteriales	Nocardioidaceae	*Aeromicrobium*	not classified
			Nocardioidaceae	*Friedmanniella*	*Friedmanniella sagamiharensis*
			Nocardioidaceae	*Nocardioides*	*Nocardioides dokdonensis*
			Propionibacteriaceae	*Microlunatus*	*Microlunatus phosphovorus*
			Pseudonocardiaceae	*Pseudonocardia*	not classified
		Streptomycetales	Streptomycetaceae	*Streptomyces*	not classified
Alphaproteobacteria	Rickettsiales	Rickettsiaceae	Rickettsieae	*Rickettsia*	not classified
Bacteria	Acidobacteria	Vicinamibacteria	Vicinamibacteraceae	*Luteitalea*	*Luteitalea pratensis*
Bacteroidetes	Bacteroidia	Bacteroidales	Paludibacteraceae	*Paludibacter*	*Paludibacter propionicigenes*
			Porphyromonadaceae	*Porphyromonas*	not classified

	Cytophagia	Cytophagales	Cytophagaceae	*Spirosoma*	*Spirosoma* sp.
			Hymenobacteraceae	*Hymenobacter*	*Hymenobacter* sp.
				Pontibacter	not classified
	Flavobacteriia	Flavobacteriales	Flavobacteriaceae	*Flavobacterium*	*Flavobacterium nackdongense*
			Weeksellaceae	*Chryseobacterium*	*Chryseobacterium glaciei*
				Cloacibacterium	*Cloacibacterium normanense*
	Sphingobacteriia	Sphingobacteriales	Sphingobacteriaceae	*Mucilaginibacter*	not classified
				Pedobacter	*Pedobacter cryoconitis*
				Sphingobacterium	*Sphingobacterium* sp.
Cyanobacteria	Cyanobacteria	Nostocales	Nostocaceae	*Anabaena*	*Anabaena* sp.
Firmicutes	Bacilli	Bacillales	Bacillaceae	*Bacillus*	not classified
				Geobacillus	not classified
				Terribacillus	*Terribacillus goriensis*
			Bacillales	*Gemella*	not classified
			Listeriaceae	*Listeria*	not classified
			Paenibacillaceae	*Paenibacillus*	*Paenibacillus cellulositrophicus*
			Staphylococcaceae	*Staphylococcus*	*Staphylococcus auricularis*
		Lactobacillales	Aerococcaceae	*Aerococcus*	not classified
			Carnobacteriaceae	*Carnobacterium*	not classified
			Carnobacteriaceae	*Dolosigranulum*	*Dolosigranulum pigrum*
			Enterococcaceae	*Enterococcus*	not classified
				Vagococcus	not classified
			Lactobacillaceae	*Lactobacillus*	*Lactobacillus iners*
					Lactobacillus jensenii
				Leuconostoc	*Leuconostoc carnosum*
				Limosilactobacillus	*Limosilactobacillus fermentum*
				Weissella	*Weissella ceti*
			Streptococcaceae	*Lactococcus*	*Lactococcus lactis*
				Streptococcus	not classified

	Clostridia	Eubacteriales	Lachnospiraceae	*Lachnoclostridium*	not classified
			Peptostreptococcaceae	*Peptostreptococcaceae*	*Peptostreptococcaceae* bacterium oral taxon 929
	Negativicutes	Selenomonadales	Selenomonadaceae	*Selenomonas*	not classified
		Veillonellales	Veillonellaceae	*Dialister*	not classified
				Veillonella	not classified
	Tissierellia	Tissierellales	Peptoniphilaceae	*Anaerococcus*	not classified
				Finegoldia	*Finegoldia magna*
				Peptoniphilus	*Peptoniphilus harei*
Fusobacteria	Fusobacteriia	Fusobacteriales	Fusobacteriaceae	*Fusobacterium*	*Fusobacterium nucleatum*
Nitrospirae	Nitrospira	Nitrospirales	Nitrospiraceae	*Nitrospira*	not classified
Proteobacteria	Alphaproteobacteria	Caulobacterales	Caulobacteraceae	*Brevundimonas*	*Brevundimonas* sp.
				Caulobacter	not classified
		Pelagibacterales	Pelagibacterales	*Candidatus* Fonsibacter	*Candidatus* Fonsibacter ubiquis
		Rhizobiales	Bradyrhizobiaceae	*Bosea*	not classified
			Hyphomicrobiaceae	*Hyphomicrobium*	not classified
			Methylobacteriaceae	*Methylobacterium*	not classified
		Rhodobacterales	Rhodobacteraceae	*Paracoccus*	not classified
		Rickettsiales	Anaplasmataceae	*Wolbachia*	*Wolbachia* endosymbiont of *Cimex lectularius*
		Sphingomonadales	Sphingomonadaceae	*Novosphingobium*	not classified
				Sphingobium	not classified
				Sphingomonas	not classified
	Betaproteobacteria	Burkholderiales	Burkholderiaceae	*Cupriavidus*	not classified
				Lautropia	*Lautropia mirabilis*
				Paraburkholderia	*Paraburkholderia fungorum*
				Ralstonia	not classified
			Comamonadaceae	*Delftia*	not classified
			Oxalobacteraceae	*Collimonas*	not classified
			Oxalobacteraceae	*Duganella*	*Duganella* sp.

			Oxalobacteraceae	*Janthinobacterium*	not classified
			Oxalobacteraceae	*Massilia*	*Massilia oculi*
		Neisseriales	Neisseriaceae	*Neisseria*	*Neisseria elongata*
	Epsilonproteobacteria	Campylobacterales	Campylobacteraceae	*Campylobacter*	not classified
	Gammaproteobacteria	Enterobacterales	Erwiniaceae	*Erwinia*	*Erwinia persicina*
				Pantoea	*Pantoea agglomerans*
			Morganellaceae	*Prevotella*	not classified
				Providencia	not classified
			Yersiniaceae	*Serratia*	not classified
		Methylococcales	Methylothermaceae	*Methylothermaceae*	*Methylomonas clara*
		Oceanospirillales	Oceanospirillaceae	*Marinomonas*	not classified
		Pasteurellales	Pasteurellaceae	*Haemophilus*	*Haemophilus parainfluenzae*
		Pseudomonadales	Moraxellaceae	*Acinetobacter*	*Acinetobacter calcoaceticus*
					Acinetobacter larvae
					Acinetobacter schindleri
					Acinetobacter ursingii
				Moraxella	*Moraxella catarrhalis*
					Moraxella osloensis
			Pseudomonadaceae	*Pseudomonas*	*Pseudomonas cichorii*
					Pseudomonas sp.
					Pseudomonas synxantha
					Pseudomonas viridiflava
		Vibrionales	Vibrionaceae	*Vibrio*	not classified
		Xanthomonadales	Rhodanobacteraceae	*Rhodanobacter*	*Rhodanobacter denitrificans*
			Xanthomonadaceae	*Stenotrophomonas*	*Stenotrophomonas maltophilia*
					Stenotrophomonas rhizophila

	cellular organisms	Bacteria	Bacteria incertae sedis	Bacteria candidate phyla	*Candidatus* Saccharibacteria
Bacteria	Bacteria incertae sedis	Bacteria candidate phyla	Candidatus Dependentiae	unclassified Candidatus Dependentiae	*Candidatus* Dependentiae bacterium

Table 5S. Results of performed statistical tests for appropriateness and fit of models considered for Microbial composition analyses. For zero-inflation and nonparametric dispersion, tests obtained p values (first line), as well as statistic (second line), are presented. For checking the fit of the model pseudo R2 determination coefficient parameter is reported for each model

	**Genus**			**Species**		
	hNB GLMM	NB GLMM	hNB GLM	hNB GLMM	NB GLMM	hNB GLM
Zeros Inflation	0.468<br>1.0008	0.008<br>1.0147	0.392<br>1.0085	0.472<br>1.0002	$<2.2*10^{-16}$<br>1.1451	0.476<br>1.0016
Dispersion	0.048<br>8.2004	$<2.2*10^{-16}$<br>$1.09*10^{-12}$	0.256<br>1.4932	0.424<br>1.2926	0.04<br>6.3178	$<2.2*10^{-16}$<br>$9.5*10^{-27}$
R2 Nakagawa	0.6901	0.0247	0.0092	0.6933	0.1287	0.0123

Table 6S. Significant differences between individual pairs of levels of variables calculated separately for CLB development stage, plant host, and locations using Multiple Comparisons of Means (Tukey Contrasts). Significant codes for p value: ***0.001, **0.01, *0.05, ˙0.1, 1

**Variable**	**Pairs of levels**	**p value**	
		**Genus**	**Species**
**Developmental stage**	larva – imago	1.25e-12***	0.00399 **
**Cereal plant host**	winter barley – spring barley	0.99998	0.88185
	oat – spring barley	0.86705	0.99999
	spring wheat – spring barley	0.88816	0.99875
	winter wheat – spring barley	<0.001***	< 0.001 ***
	triticale – spring barley	0.43375	0.45828
	rye – spring barley	0.80702	0.83687
	oat – winter barley	0.93383	0.96600
	spring wheat – winter barley	0.92824	0.97679
	winter wheat – winter barley	< 0.001 ***	0.15324
	triticale – winter barley	0.80281	0.99945
	rye – winter barley	0.82837	0.33970
	spring wheat – oat	0.26078	1.00000

	winter wheat – oat	0.00128 **	0.00307 **
	triticale – oat	0.99661	0.78425
	rye – oat	0.31465	0.87517
	winter wheat – spring wheat	< 0.001 ***	< 0.001 ***
	triticale – spring wheat	0.05538 .	0.72425
	rye – spring wheat	0.99924	0.58524
	triticale – winter wheat	0.01409 *	0.26532
	rye – winter wheat	< 0.001 ***	< 0.001 ***
	rye – triticale	0.02339 *	0.02431 *
**Location**	Winna Góra – Kościelna Wieś	0.000483 ***	0.057293 .
	Zybiszów – Kościelna Wieś	< 1e-04 ***	0.000544 ***
	Zybiszów – Winna Góra	0.000114 ***	0.375375

Table 7S. The results of the statistical analysis presented as the p-values of the tests used to characterize the alpha and beta diversity bacterial genera and species associated with CLB depending on the locations. Significant codes for p-value: ***0.001, **0.01, *0.05, ˙0.1, 1

**variable: location**		
**test**	**p-value**	
	**genus level**	**species level**
***α*-diversity**		
Kruskal Wallis	$3.11*10^{-5}$	$2.63*10^{-6}$
***β*-diversity** (PERANOVA)		
Jaccard	0.001***	0.018*
Bray	0.001***	0.020*
Chao	0.001***	0.002**

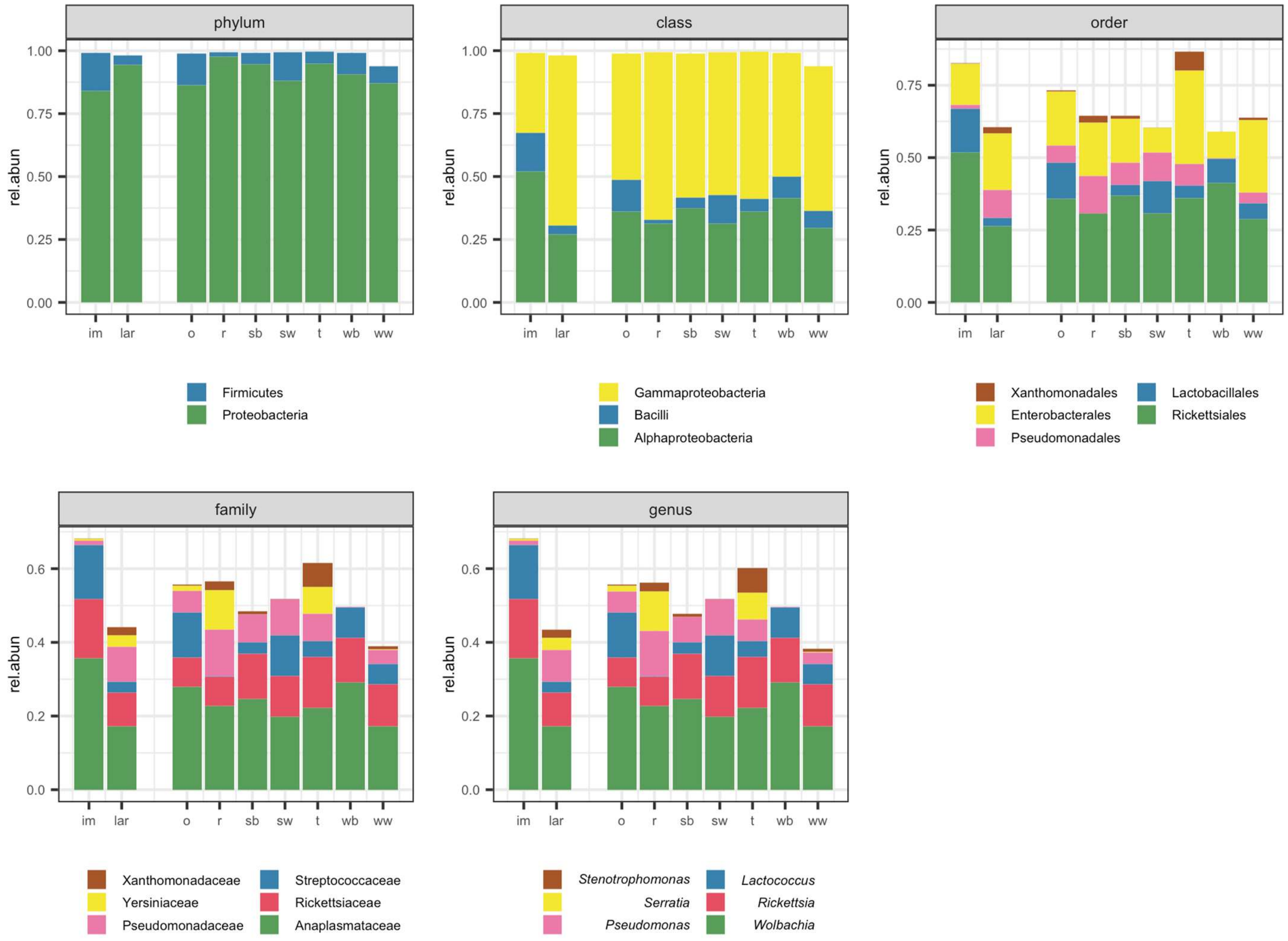

Figure 1S. CLB-associated bacteria distribution at phylum, class, order, family, and genus level depending on the variables: insect`s developmental stage (imago, larva) and cereal plant host (spring barley, winter barley, spring wheat, winter wheat, oat, rye, and triticale). The mean value of relative abundance for rank taxa from Top Biome are included in the graph. Abbreviations: im-imago, lar-larva, sb-spring barley, wb-winter barley, o-oat, sw-spring wheat, ww-winter wheat, t-triticale, r-rye

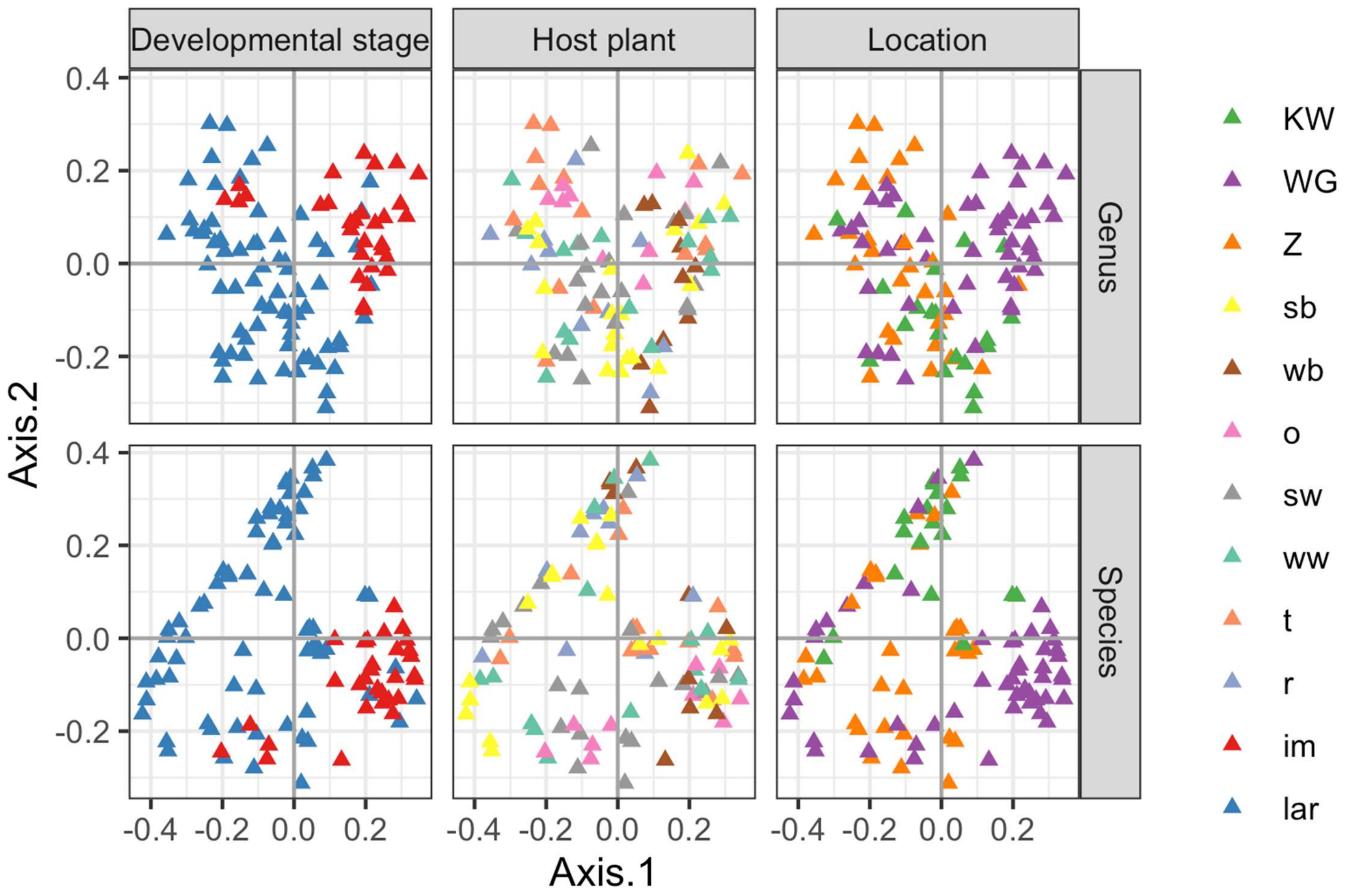

Figure 2S. The visualization of PCoA based on insect's developmental stages, plant host and location from which CLB were collected. Abbreviations: KW-Kościelna Wieś, WG-Winna Góra, Z-Zybiszów, sb-spring barley, wb-winter barley, o-oat, sw-spring wheat, ww-winter wheat, t-triticale, r-rye, im-imago,lar-larva

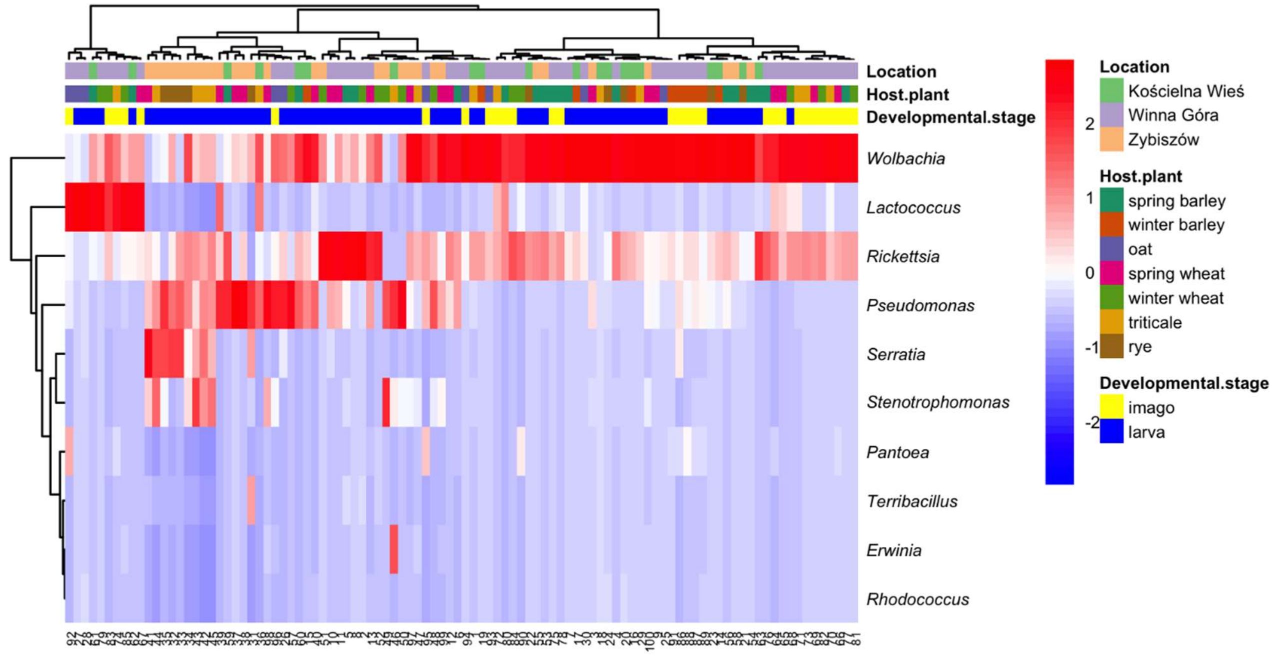

Figure 3S. The heatmap based on hierarchical clustering with Ward method for the relative abundance of the 10 Top Biome CLB associated bacteria genera in relation to the CLB developmental stage (imago, larva), host plant (winter wheat, spring wheat, winter barley, spring barley, oat, triticale, rye) and location (Winna Góra, Kościelna Wieś, Zybiszów) from which the insects were collected

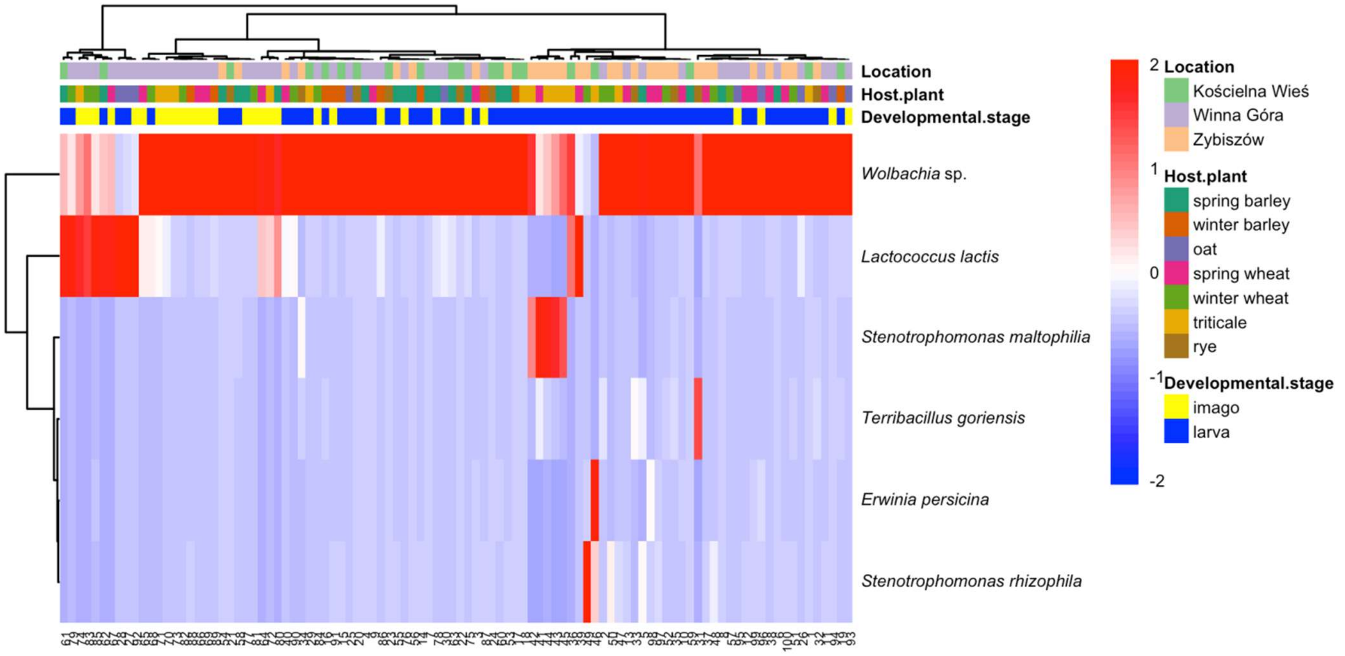

Figure 4S. The heatmap based on hierarchical clustering with method Ward for the relative abundance of the 6 Top Biome CLB associated bacteria species in relation to the CLB developmental stage (imago, larva), host plant (winter wheat, spring wheat, winter barley, spring barley, oat, triticale, rye) and location (Winna Góra, Kościelna Wieś, Zybiszów) from which the insects were collected

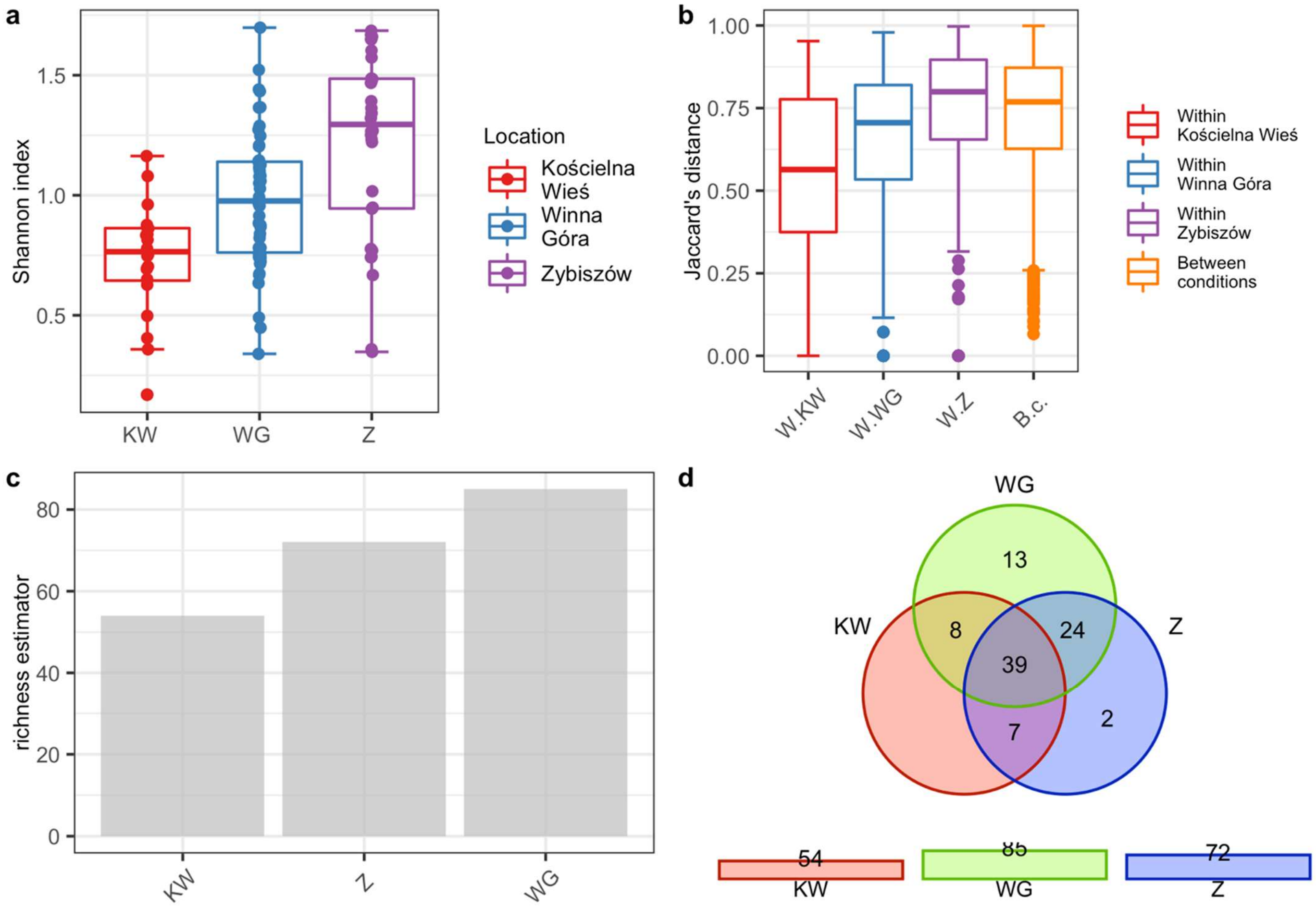

Figure 5S. Diversity and richness of CLB bacterial community at the genus level depending on the locations: a) alpha-diversity (Shannon index); b) beta diversity (Jaccard distance); c) level of richness (jacknife

coefficient); d) a number of shared genera between imagoes and larvae (Venn`s diagram). Abbreviations: WG - Winna Góra, KW - Kościelna Wieś, Z - Zybiszów, W.WG - within Winna Góra, W.KW - within Kościelna Wieś, W.Z - within Zybiszów, B.c - between conditions

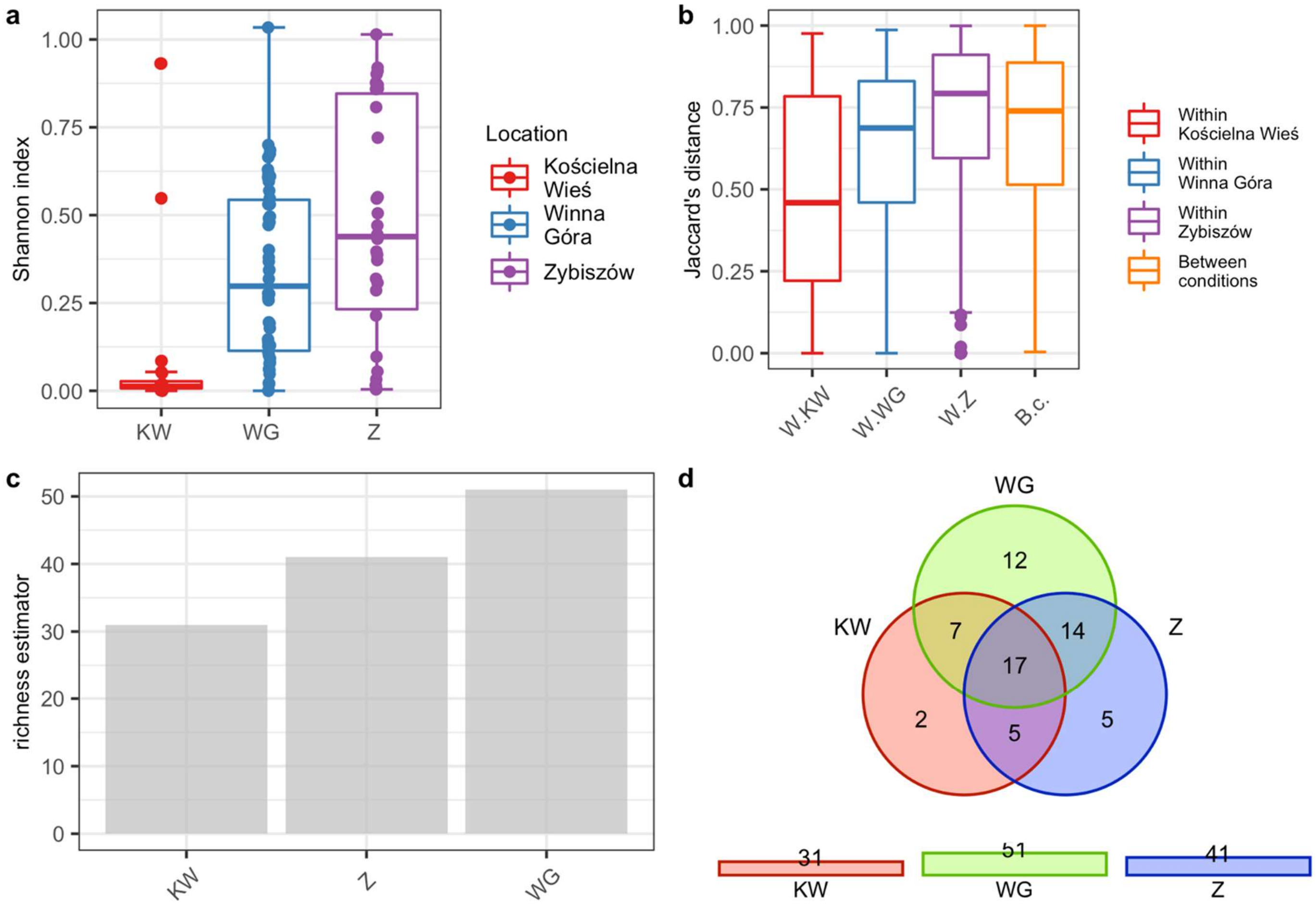

Figure 6S. Diversity and richness of CLB bacterial community at the species level depending on the locations: a) alpha-diversity (Shannon index); b) beta diversity (Jaccard distance); c) level of richness (jacknife coefficient); d) a number of shared species between imagoes and larvae (Venn`s diagram). Abbreviations: WG - Winna Góra, KW - Kościelna Wieś, Z - Zybiszów, W.WG - within Winna Góra, W.KW - within Kościelna Wieś, W.Z - within Zybiszów, B.c - between conditions

Table 8S. List of shared bacteria species observed in at least one sample among insects for the variable CLB developmental stage and cereal plant host from which insects were collected. Bacterial species marked with an asterisks belong to the top biome

	**CLB developmental stage**		**cereal plant host**	
	**Genus**	**Species**	**Genus**	**Species**
1	*Acinetobacter*	*Acinetobacter calcoaceticus*	*Acinetobacter*	*Lactococcus lactis****
2	*Actinomyces*	*Acinetobacter schindleri*	*Bacillus*	*Moraxella osloensis*
3	*Aerococcus*	*Anabaena* sp.	*Chryseobacterium*	*Wolbachia* sp.***
4	*Aeromicrobium*	*Candidatus Fonsibacter*	*Corynebacterium*	
5	*Anabaena*	*Chryseobacterium glaciei****	*Lactococcus****	
6	*Anaerococcus*	*Cloacibacterium normanense*	*Moraxella*	
7	*Arthrobacter*	*Erwinia persicina****	*Pantoea****	
8	*Bacillus*	*Finegoldia magna*	*Paracoccus*	
9	*Brevibacterium*	*Fusobacterium nucleatum*	*Pseudomonas****	

10	*Brevundimonas*	*Haemophilus parainfluenzae*	*Rhodococcus****	
11	*Campylobacter*	*Hymenobacter* sp.	*Rickettsia****	
12	*Candidatus*	*Lactobacillus iners*	*Serratia****	
13	*Carnobacterium*	*Lactococcus lactis****	*Sphingomonas*	
14	*Chryseobacterium*	*Lautropia mirabilis*	*Staphylococcus*	
15	*Cloacibacterium*	*Lawsonella clevelandensis*	*Stenotrophomonas****	
16	*Corynebacterium*	*Luteitalea pratensis*	*Streptococcus*	
17	*Cupriavidus*	*Massilia oculi*	*Wolbachia****	
18	*Erwinia****	*Microlunatus phosphovorus*		
19	*Finegoldia*	*Moraxella catarrhalis*		
20	*Flavobacterium*	*Moraxella osloensis*		
21	*Fusobacterium*	*Neisseria elongata*		
22	*Haemophilus*	*Paludibacter propionicigenes*		
23	*Hymenobacter*	*Pantoea agglomerans*		
24	*Hyphomicrobium*	*Paraburkholderia fungorum*		
25	*Janthinobacterium*	*Rhodanobacter denitrificans*		
26	*Lachnoclostridium*	*Sanguibacter keddiei*		
27	*Lactobacillus*	*Stenotrophomonas rhizophila****		
28	*Lactococcus****	*Wolbachia* sp.***		
29	*Lautropia*			
30	*Lawsonella*			
31	*Luteitalea*			
32	*Marinomonas*			
33	*Massilia*			
34	*Methylobacterium*			
35	*Microlunatus*			
36	*Moraxella*			
37	*Mucilaginibacter*			
38	*Neisseria*			
39	*Nocardioides*			
40	*Novosphingobium*			
41	*Paenibacillus*			
42	*Paludibacter*			
43	*Pantoea****			
44	*Paraburkholderia*			
45	*Paracoccus*			
46	*Pontibacter*			
47	*Prevotella*			
48	*Pseudomonas****			
49	*Pseudonocardia*			
50	*Ralstonia*			
51	*Rhodanobacter*			
52	*Rhodococcus****			
53	*Rickettsia****			
54	*Sanguibacter*			
55	*Selenomonas*			
56	*Serratia****			
57	*Sphingobium*			
58	*Sphingomonas*			
59	*Spirosoma*			
60	*Staphylococcus*			
61	*Stenotrophomonas****			
62	*Streptococcus*			
63	*Veillonella*			
64	*Vibrio*			
65	*Weissella*			

66	*Wolbachia****			

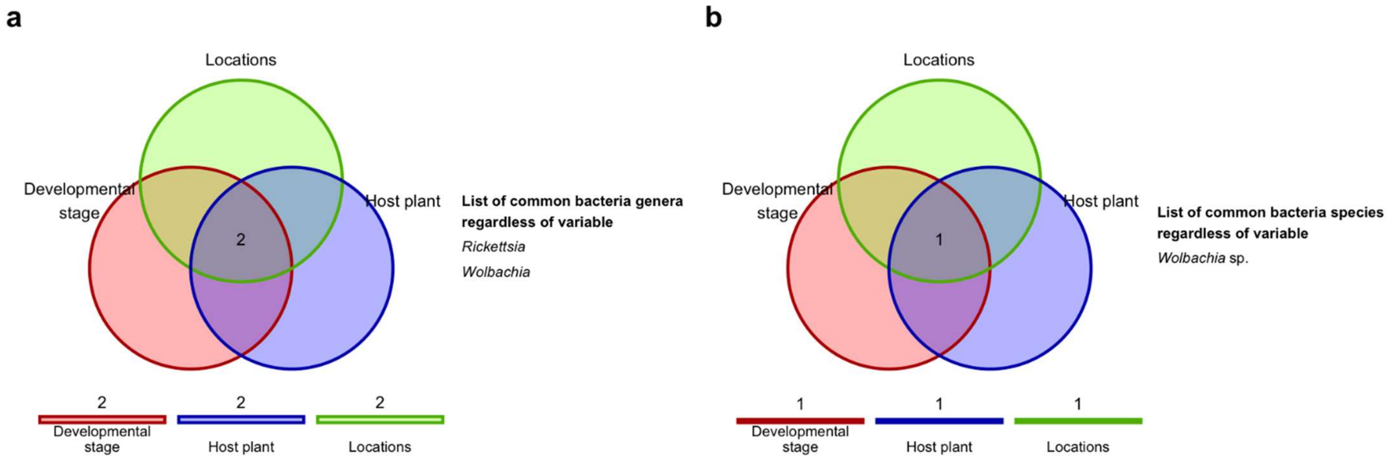

Figure 7S. The core microbiome of CLB insects is described as the number of a) genera and b) species of bacteria shared by the majority (at least 90%) of CLB insects in each group regardless of the all tested variables (developmental stage, cereal plant host and locations), visualized by the Venn diagram
